# Strong coupling of Asian Monsoon and Antarctic climates on sub-orbital timescales

**DOI:** 10.1038/srep32995

**Published:** 2016-09-08

**Authors:** Shitao Chen, Yongjin Wang, Hai Cheng, R. Lawrence Edwards, Xianfeng Wang, Xinggong Kong, Dianbing Liu

**Affiliations:** 1 Key Laboratory of Virtual Geographic Environment, Nanjing Normal University, Nanjing 210023, China; 2 Jiangsu Center for Collaborative Innovation in Geographic Information Resource Development and Application, Nanjing 210023, China; 3 Institute of Global Environmental Change, Xi’an Jiaotong University, Xi’an 710049, China; 4 Department of Geology and Geophysics, University of Minnesota, Minneapolis, Minnesota 55455, USA; 5 Earth Observatory of Singapore, Nanyang Technological University, Nanyang Avenue 639798, Singapore; 6 State Key Laboratory Cultivation Base of Geographical Environment Evolution, Jiangsu Province, Nanjing 210023, China

## Abstract

There is increasing evidence that millennial-scale climate variability played an active role on orbital-scale climate changes, but the mechanism for this remains unclear. A ^230^Th-dated stalagmite *δ*^18^O record between 88 and 22 thousand years (ka) ago from Yongxing Cave in central China characterizes changes in Asian monsoon (AM) strength. After removing the 65°N insolation signal from our record, the *δ*^18^O residue is strongly anti-phased with Antarctic temperature variability on sub-orbital timescales during the Marine Isotope Stage (MIS) 3. Furthermore, once the ice volume signal from Antarctic ice core records were removed and extrapolated back to the last two glacial-interglacial cycles, we observe a linear relationship for both short- and long-duration events between Asian and Antarctic climate changes. This provides the robust evidence of a link between northern and southern hemisphere climates that operates through changes in atmospheric circulation. We find that the weakest monsoon closely associated with the warmest Antarctic event always occurred during the Terminations. This finding, along with similar shifts in the opal flux record, suggests that millennial-scale events play a key role in driving the deglaciation through positive feedbacks associated with enhanced upwelling and increasing CO_2_.

Millennial-scale climate changes over the several recent ice-age cycles have been documented in records of deep-sea sediments (e.g., ref. 1), Antarctic ice cores^2^,^3^ and Chinese speleothems^4^,^5^. Several studies on the link between millennial-scale events and ice-age Terminations^4^,^6^ have suggested that these events may play an active role in driving the deglaciation. The mechanism involves positive feedbacks associated with CO_2_ increase via the oceanic bipolar seesaw and/or the southward displacement of the Southern Hemisphere (SH) westerlies^4^,^6^,^7^,^8^,^9^. Available evidences for the phase relationship and transferring fashion of the inter-hemispheric millennial-scale changes primarily come from studies of polar ice cores^10^,^11^. Recently, the highest resolution Antarctic ice core from the West Antarctic Ice Sheet (WAIS) Divide (WDC)^12^ established a north-to-south directionality and displayed that the Antarctic temperature maxima occurred ~200 years after Greenland warming transitions. This finding points to an inter-hemispheric coupling by oceanic processes. However, Landais *et al*. (2015; ref. 13) argued that abrupt northern warming was concomitant with onset of southern cooling by use of stacks of Greenland and Antarctic ice core records which were bypassed apparent ‘noises’ in the original records. This suggests an atmospheric teleconnection between hemispheric climates. The uncertainties of the phase relationship between millennial-scale climate events in the two hemispheres greatly complicate interpretations of the forcing mechanisms for ice-age cycles. High-resolution records from Antarctic ice cores^2^,^3^ and Chinese speleothems^4^,^5^ can enable us to address the debated issue. Our research begins with the last glacial and is subsequently extrapolated to the last two glacial-interglacial cycles.

Here we report a new stalagmite-based record from Yongxing Cave (see Supplementary Fig. S1) on the eastern slope of Shengnongjia Mountain, Hubei Province, central China, where the climate is dominated by the AM. Yongxing Cave is poorly ventilated and the humidity in the cave reaches about 100%. Mean annual precipitation and mean annual temperature are ~1,000 mm and ~12 °C at the cave site, respectively. The temperature inside the cave is about 14 °C. Rainfall in this zone is controlled by the seasonal changes in solar radiation which result in a migration of the intertropical convergence zone (ITCZ) and changes in monsoon intensity.

Three stalagmite samples (see Supplementary Fig. S2) were U-Th dated, and their respective calcite *δ*^18^O time series are reported here (see Supplementary Tables S1 and S2). The Yongxing *δ*^18^O record covers a period from 88 to 22 ka BP (before present, present = AD 1950). There is significant overlap among the three stalagmite *δ*^18^O records over their contemporaneous growth periods (57.5–34.8 ka BP and 65.0–61.4 ka BP), suggesting that the *δ*^18^O variations were primarily caused by climate changes at the cave site. Following the reasoning in ref. 14, we interpret lighter *δ*^18^O values to reflect stronger AM intensity, and vice versa.

Striking similarity between Yongxing Cave and the previously-published Hulu Cave record^14^ (see Fig. 1) suggests regional coherence of *δ*^18^O variations. Our new record allows for a better constraint on the AM events that occur on time scales from millennia to sub-millennia and shorter, such as the double peak characterizing Dansgaard-Oeschger event (DO) 15. Furthermore, the event DO 18, found in Greenland ice core records (e.g., ref. 15), is indistinct in the low-resolution Hulu record^14^, but obviously exhibited with ~2% enriched in *δ*^18^O at ~64.5 ka BP in the Yongxing record. The last glacial AM has been extended back to 88 ka BP in the Yongxing record, which covers the full MIS5a with interglacial conditions, characterized with fine-*δ*^18^O-structure for DO 20 and 21. Compared with the Hulu Cave record^14^, our new record is marked by more robust chronology (72 ^230^Th dates with a typical uncertainty of ~200 years), higher resolution (~70 years on average) and a larger amplitude (~4% on *δ*^18^O). Therefore, Yongxing Cave record can be used as a new reference for quantifying the timing and pattern of monsoonal climate events during the last glacial period.

Figure 1 provides a comparison between Greenland NGRIP ice core^15^ and Yongxing Cave *δ*^18^O records. The NGRIP record is on the GICC05 chronology^16^ constructed by counting annual layers for time younger than 60 ka BP. In general, timing of the strong monsoon events in the Yongxing record agrees well, within dating uncertainties, with their equivalent DO events of Greenland *δ*^18^O record on the GICC05 chronology. This observation strongly argues for an in-phase relationship of millennial-scale climatic variations within the northern hemisphere as noted by early studies^5^,^14^. For time older than 60 ka BP, an age offset between DO and monsoon events irregularly becomes large. Especially for events 19 and 20, Yongxing record is ~1.5 ka younger than the NGRIP record on ss09sea timescale^17^. The two events in the Yongxing record constrained by 6 ^230^Th dates with a mean error of 300 years match well with the Sanbao record^4^,^5^. We speculate that the age offset is possibly derived from the uncertainties of Greenland timescale^18^.

Despite good correlation between the Chinese cave and Greenland records for millennial-scale changes, the Hulu record appears to resemble Antarctic temperature variability at certain stadial-interstadial transitions^19^. Statistical analyses further showed that 70 percent of the variance in the Hulu record is reflected in the Antarctic time series in the late MIS3 (ref. 20). As demonstrated previously^4^,^5^,^14^, AM changes were predominated by both Northern Hemisphere summer insolation (NHSI) and millennial-scale climate forcing. Thus, after removal of the NHSI signal, the detrended speleothem *δ*^18^O record (Δ*δ*^18^O; see Methods) could better characterize the weak monsoon intervals (WMI) (ref. 7) (see Fig. 2). Indeed, the Δ*δ*^18^O variations show high resemblance to Antarctic temperature variations as recorded by WDC ice core^12^, the highest resolution Antarctic record. The period of 70–35 ka BP was used for comparison because it brackets the Antarctic warming events (A) 1–4, and may be less impacted by orbital changes (i.e., global ice volume). This resemblance was also supported by the Antarctic temperature profiles from Byrd, EPICA Dronning Maud Land (EDML), and EPICA Dome C (EDC) ice-core records^2^,^10^,^11^, located in the Pacific, Atlantic and Indian Ocean sectors, respectively (see Fig. 2).

It is therefore necessary to remove the impact of different orbital backgrounds to make millennial-scale changes stand out in these records, when extending the comparison into the last two glacial-interglacial cycles. We constructed a composite speleothem *δ*^18^O record (see Methods and Fig. 3a) and removed the NHSI signal. For the Antarctic climate change, we decompose the millennial-scale events by removal of the LR04 marine stack^21^, an indicator of ice-volume signal, from EDC ice core isotopic record^2^ on the AICC2012 chronology^22^ (see Methods and Supplementary Fig. S3). As a result, 21 warming events in the Antarctic EDC residual records (Δ*δ*D) have been matched to their unique counterparts in the cave residual records (Δ*δ*^18^O) (see Fig. 3b). In particular, different patterns of events (e.g., in the three Terminations and MIS6) in the raw records are now essentially the same. Similar results are shown in Antarctic Vostok ice core records^3^ (see Supplementary Fig. S4). Both data further indicate that the general nature of these changes is reproducible for the last two glacial-interglacial cycles. We name these events A1 to A9 for the last glacial-interglacial cycle, B1 to B9 for the penultimate cycle, and giant events G1 to G3 for the last three Terminations (see Fig. 3b).

A comparison of Greenland and Antarctic ice core *δ*^18^O records, synchronized by the atmospheric methane record, indicates that Antarctica temperature gradually rise when Greenland is cold, whereas cooling starts in the south as soon as the northern temperature jumps high^10^,^11^. However, such an observation precludes a precise determination of the lead-lag relationship between bipolar climate changes^23^. One of the main reasons is that different characteristics of Greenland and Antarctic ice-core records lead to weaker expressions of the northern signal in the Antarctic record. Here, the extreme similarity of the cave Δ*δ*^18^O and Antarctic Δ*δ*D (see Fig. 3b) is a convincing basis for inferring such a relationship. More specifically, between 66 and 56 ka BP, the caveΔ**^18^O and Antarctic methane faithfully record a sequence of major oscillations that each lasted for 1 ka or less (see Supplementary Fig. S5). The synchronization uncertainty of the cave *δ*^18^O and Antarctic methane^12^, ranging from 200 to 300 years, is adequate to validate the basic correlation of the inter-hemispheric climate signals at longer suborbital wavelengths (~7 ka) (i.e., A4). The excellent correlations, even for small oscillations (i.e., DO 17 and 18), permit a synchronization of no more than 300 years (see Supplementary Fig. S5).

Based on our correlation analysis, the warmest intervals in Antarctica occurred synchronously with the weakest monsoon intervals during the last three deglaciations, respectively (Yellow bars in Fig. 3). Marine records from the SH have well defined sea surface temperature (SST) maxima that occur thousands of years before the *δ*^18^O minima in the same cores^24^. If the Antarctic warm peaks are anchored to the SST maxima, the WMIs in Chinese caves and the warm peaks in Antarctica end at the same time. This observation confirms a recent suggestion^25^ of a near synchrony of Antarctic and Greenland transitions spanning the deglaciation within a dating uncertainty of 200 years. Our results support the idea that the North Atlantic was coldest and Antarctica was warmest at the same time^26^.

Despite a linear relationship between cave Δ*δ*^18^O and EDC Δ*δ*D, not every WMI is as distinct as the Antarctic temperature maximum. The most obvious discrepancy occurs during 225–195 ka BP (see Fig. 3b), probably due to the age uncertainties and temporal resolutions of the records. The correlation between cave Δ*δ*^18^O and EDC Δ*δ*D in the last glacial-interglacial cycle (r = 0.76) is more significant than that in the penultimate cycle (r = 0.51). Because the chronological control and temporal resolution are much better for the last cycle than for the penultimate glacial-interglacial cycle for all the records, we propose that higher quality data could make them closer to the same on sub-orbital timescales.

This linear correlation we observed here cannot be explained simply by the bipolar seesaw model. The thermal seesaw concept^27^ could account for a linkage between the different characteristics inherent in Greenland and Antarctic events, but time is required to transmit the northern signal to the south as a result of thermal inertia in the Southern Ocean. When fresh water was released into the North Atlantic, the AM climates were influenced both by cooling northern high-latitude climate via the westerly wind belt and by the gradual warming in the south^20^,^28^. This combined effect could not produce a linear relationship between the two hemispheres (see Fig. 3b). Secondly, the oceanic seesaw model is unlikely to have operated across all timescales, from the short duration of a DO event, to a Bond cycle, to the dramatic oscillations at glacial terminations (Fig. 3) that are far beyond the periodicity of oceanic circulation (<7 ka) (ref. 27). Modeling experiments^29^ also demonstrated inconsistency with the simple bipolar seesaw paradigm and called for a more active role of atmospheric circulation.

Antarctic warming events are clearly captured by dust fluxes, indicating that these events were associated with changes in atmospheric dust transport^30^. The cave Δ*δ*^18^O also shows strong resemblance to Antarctic EDC dust record (see Supplementary Fig. S5), suggesting that Antarctic temperature is related to latitudinal shifts in the mean position of the SH westerly wind belt and the ITCZ. This is consistent with present interannual climate variability, which points to a fast teleconnection between changes in low-latitude atmospheric circulation and Antarctic temperature^31^. The dejumped dust record in Greenland was found to match Antarctic climate variability^19^, suggesting that abrupt climate change emerging from Greenland was associated with large-scale changes in atmospheric transport. In this study, the inter-hemispheric seesaw pattern defined by the cave Δ*δ*^18^O and Antarctic Δ*δ*D supports a reorganization of atmospheric circulation^28^,^32^ in addition to thermal seesaw scheme^27^.

Figure 3 shows larger-amplitude millennial-scale events at the glacial Terminations (G1-G3) in contrast to those of other millennial-scale climate changes (A1-A9 and B1-B9). An opal flux record from the Southern Ocean shows two intervals of enhanced upwelling that are concurrent with the two intervals of rising atmospheric CO_2_ during the last deglaciation^9^. The dominant features of these upwelling records are similar to the double-peak structure observed in cave Δ*δ*^18^O and in Antarctic Δ*δ*D (Fig. 4a,b). A similar feature was detected in opal flux record from equatorial Pacific sediments^33^ for the past 260 ka (Fig. 4c). Equatorial opal accumulation rates sustained relatively low values over much of the record and were punctuated by large increases in opal centered on Terminations I and II, as well as prior to Termination III. Large nutrient supplies in the Equatorial Pacific mainly originate from Subantarctic Mode Water during the Terminations^33^. These lines of evidence suggest that millennial-scale events could act as a trigger, via atmospheric teleconnections, to push the ITCZ and SH westerly southward, eventually enhancing the wind-driven upwelling and rising atmospheric CO_2_ levels that contributed to deglaciation^8^,^9^.

Our results provide a new insight into the relationship between the millennial events and the associated deglaciations. Previous studies proposed that the Antarctic warming events were indistinguishable from the beginnings of terminations in terms of shape and rate^34^. Here, our finding indicates that the giant events (G1-G3) during the terminations have twice the intensity of those in other periods (A1-A9 and B1-B9). The unusually large shifts far exceeded the proposed threshold above which the Antarctic warming events would develop into a full glacial termination^34^. This observation indicates that terminations could be considered as super-giant events^32^, during which the millennial events and a series of positive oceanic/atmospheric feedbacks^4^ played an important role in crossing the threshold. Given the worldwide emergence of remarkable similar millennial-scale records over the last two glacial-interglacial cycles, the pacing of this climate variability may represent a natural resonance in the climate system^1^. The AM transports huge amount of heat and moisture from northern Australia across the Indian and Pacific Ocean to the Asian continent during boreal summer. In contrast, the cold and dry winter monsoon, during boreal winter, flows across eastern Asia, and ultimately contributes to the Australian summer monsoon^35^. It implies that the monsoon was a critical “atmospheric bridge” rapidly connecting the high and low latitude climates, regardless of whether terminations are triggered by high-latitude^34^,^36^ or tropical climate^37^.

## Methods

### U-Series Dating

Three stalagmite samples, YX46, YX51 and YX55, were cut into halves along their growth axes and polished (see Supplementary Fig. S2). A total of 72 sub-samples (18 ^230^Th dates for YX46, 36 dates for YX51, and 18 dates for YX55) were drilled for ^230^Th dating (see Supplementary Table S1), which was performed at the Minnesota Isotope Laboratory on a multi-collector inductively coupled plasma mass spectrometer (MC-ICP-MS), Thermo-Finnigan Neptune, using procedures described in ref. 38. Typical errors (2σ) are less than 0.5%.

### Stable Isotope Analysis

Analytical procedures for *δ*^18^O are similar to those described in ref. 14. A total of 1346 powdered sub-samples (see Supplementary Table S2) were drilled at 0.5–1.0 mm intervals, and analyzed on a Finnigan-MAT 253 mass spectrometer equipped with a Kiel Carbonate Device III at Nanjing Normal University. Data were calibrated against standard NBS-19 and reported as *δ*^18^O (%) relative to the Vienna Pee Dee Belemnite (VPDB). Duplicate measurements of laboratory standard gave a reproducibility of 0.06% (1σ).

### The composite Chinese cave *δ* ^18^O record over the past 260 ka

**Figure 3:**
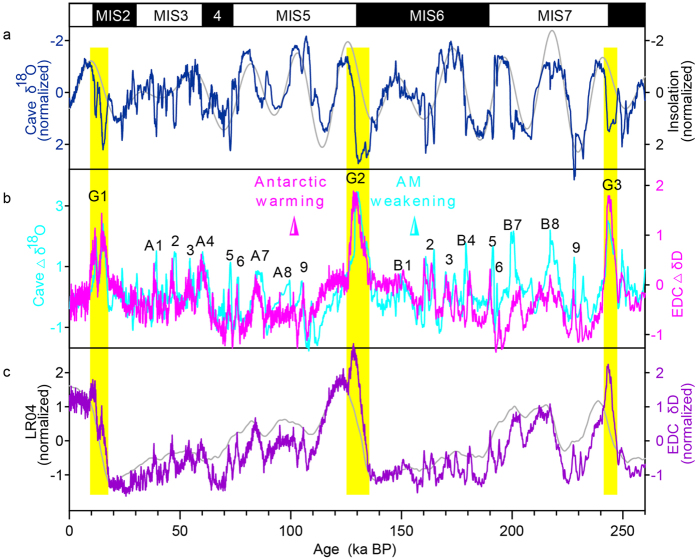
A link between orbital and millennial variability. (**a)** Composite Chinese cave *δ*^18^O record (normalized; navy blue), and the summer (21 July) insolation at 65°N (normalized; grey). (**b)** Detrended cave *δ*^18^O record (Δ*δ*^18^O; cyan) and detrended EDC *δ*D record (Δ*δ*D; magenta). (**c)** Antarctic EDC *δ*D record^2^ on the AICC2012 chronology^22^ (normalized; purple) and the LR04 marine stack^21^ (normalized; grey). A1-10 and B1-10 label the Antarctic warming events. Giant events at Terminations (G1-3) are marked with yellow bars.

The composite Chinese cave *δ*^18^O record was spliced using the Hulu^14^ (23.31–19.26 ka BP), Sanbao^4^,^5^,^39^ (19.21–0 ka BP; 261.28–78.13 ka BP) and Yongxing (78.07–23.36 ka BP) (this study) cave *δ*^18^O records (Fig. 3a). Of these, the Sanbao records typically exhibit lower average absolute *δ*^18^O values than the Hulu records by ~1.6%, and the Yongxing records by ~0.7%. These offsets mainly result from continental effects, elevation effects^5^,^14^, and/or from differences in the time of stored water at these sites. Thus, to match the Sanbao record, we subtracted the *δ*^18^O values of the Hulu records by 1.6%, and the Yongxing records by 0.7% to generate a composite record.

### Decomposition of the climatic signal

#### Data preprocessing

As described in ref. 40, data processing might amplify noise in the original record; smoothing is a simple and effective method of minimizing the effects of noise. All data presented here were gathered by applying a 3-point running mean to the evenly sampled datasets.

In order to compare and run different data sets (insolation, cave *δ*^18^O, Antarctic *δ*D and the ice-volume signal), we converted each record to a z-score by using the mean and standard deviation of each data set (i.e., zero-mean normalization). The amplitude of the insolation and the ice-volume signal were then scaled to match the cave *δ*^18^O and Antarctic *δ*D, respectively. An average data resolution of 100 years was applied to allow point-to-point alignment.

#### Removal of the long-term trend

Using the normalized data for the cave and July 21 insolation at 65°N, we obtained a set of detrended cave *δ*^18^O data (Δ*δ*^18^O) by subtracting the insolation signal from the cave *δ*^18^O records. The result is essentially similar to those described in ref. 7. Because the high-resolution Yongxing record is used here in place of the low-resolution Hulu Cave record^14^, minor differences exist in the last glaciation including timing, amplitude and pattern of the millennial-scale events.

The Antarctic EDC temperature record includes an ice-volume signal typical of “the 100 ka cycle”. For this reason, we decompose the millennial-scale variability from the EDC temperature records by removal of the LR04 marine stack, an indicator of ice-volume signal. Using the normalized data for Antarctic EDC *δ*D (ref. 2) record on the AICC2012 chronology^22^ and the LR04 marine stack^21^, we generated a set of detrended EDC data (Δ*δ*D) by subtracting the ice-volume signal from the Antarctic EDC *δ*D record. The LR04 marine stack is composed of benthic *δ*^18^O records from 57 globally distributed sites aligned by an automated graphic correlation algorithm^21^. The top 22 ka of the stack within an average dating uncertainty of 0.2 ka, dated by correlation to the ^14^C-dated benthic *δ*^18^O record^41^, is comparable with AICC2012 chronology. From 120 to 22 ka BP the stack is aligned to the high-resolution benthic *δ*^18^O record of Site MD95-2042 (ref. 42), which is dated by correlating millennial-scale features in its planktonic *δ*^18^O to ice *δ*^18^O from the Greenland ice core. Sensitivity tests show that the supposed uncertainties of LR04 chronology and AICC2012 chronology for the last glacial-interglacial cycles exhibit insignificant impact on our data processing. To test the reproducibility, we calculated the same detrended results using three SPECMAP curves described in refs 43, 44, 45, respectively, in addition to LR04 marine stack. The results are largely similar (see Supplementary Fig. S3).

## Additional Information

**How to cite this article**: Chen, S. *et al*. Strong coupling of Asian Monsoon and Antarctic climates on sub-orbital timescales. *Sci. Rep*. **6**, 32995; doi: 10.1038/srep32995 (2016).

## Supplementary Material

Supplementary Information

## Figures and Tables

**Figure 1:**
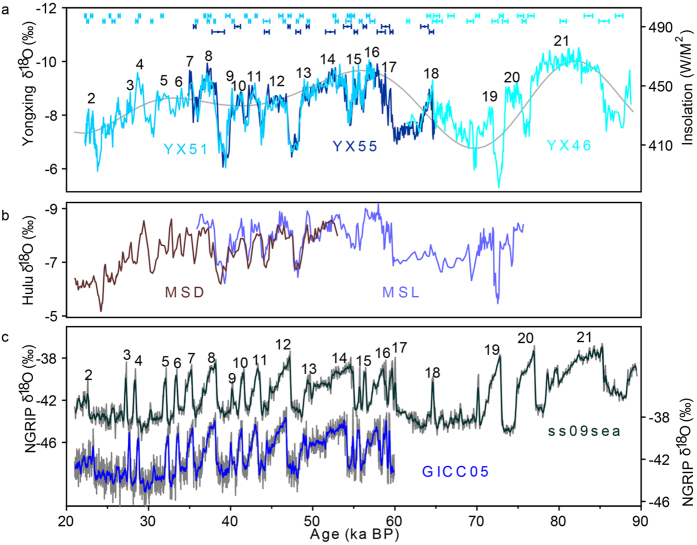
Comparison of the cave and Greenland records. (**a)** *δ*^18^O records from Yongxing Cave stalagmites (YX51, sky blue; YX55, navy blue; YX46, cyan), and the summer (21 July) insolation at 65°N (grey). ^230^Th ages and 2σ errors shown at the top are color-coded as for stalagmites. (**b)** *δ*^18^O records from Hulu Cave stalagmites^14^ (MSD, dark brown; MSL, electric blue). (**c)** *δ*^18^O records from Greenland NGRIP ice core^15^ on ss09sea timescale^17^ (black) and GICC05 timescale^16^ (blue), respectively. Numbers refer to DO events.

**Figure 2:**
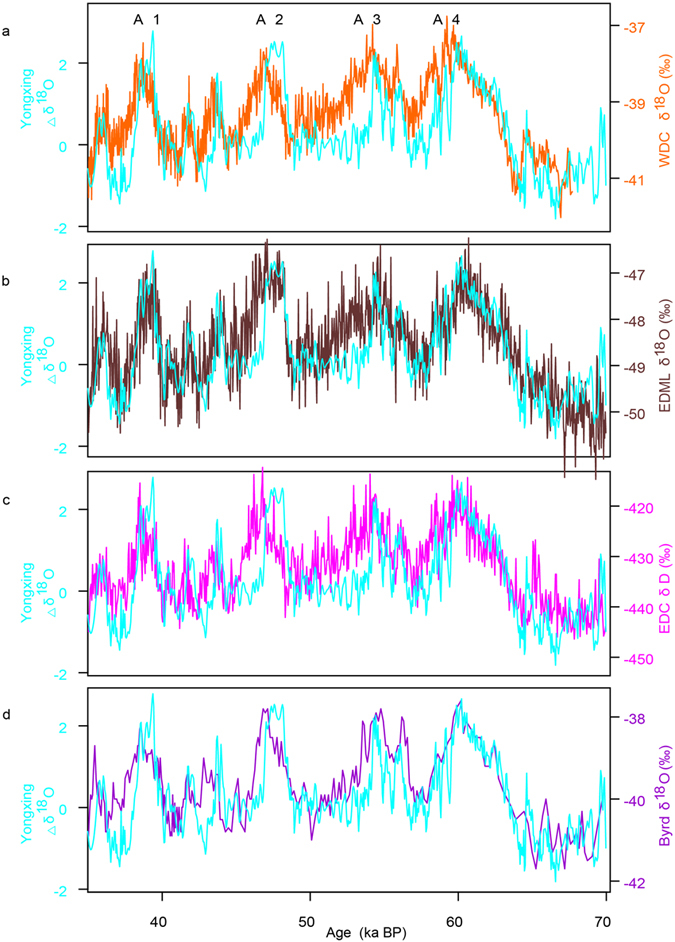
Comparison of the detrended Yongxing Cave *δ*^18^O record (Δ*δ*^18^O; cyan) with the Antarctic ice core records during 70-35 ka BP. At the millennial scale, the weak monsoon events are similar but opposite to changes in the Antarctic temperature indicated by (**a)** *δ*^18^O record from WDC (ref. 12; orange); (**b)** *δ*^18^O record from EDML (ref. 11; dark brown); (**c)** *δ*D record from EDC (ref. 2; magenta); and (**d)** *δ*^18^O record from Byrd ice core^10^ (purple). All Antarctic records are methane synchronized and given on the WD2014 chronology for the WDC ice core^12^. The Antarctic warming events, as defined in ref. 10, were denoted by A1 to A4, respectively.

**Figure 4:**
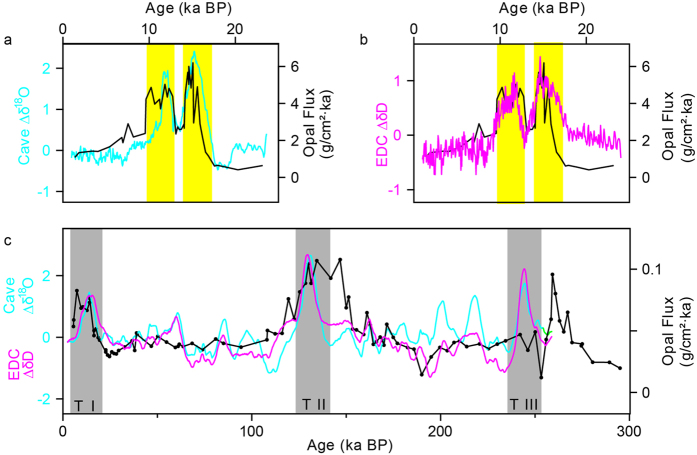
Coupling of sub-orbital timescale changes in EDC Δ*δ*D, cave Δ*δ*^18^O and ocean upwelling. (**a, b)** Cave Δ*δ*^18^O (cyan), EDC Δ*δ*D (ref. 2; magenta) and opal flux from TN057-13PC in the Southern Ocean^9^ (black) during Termination (T) I. The double-peak structures are highlighted by yellow bars. (**c)** Cave Δ*δ*^18^O (cyan) and EDC Δ*δ*D (magenta) as in Fig. 3b but 99-point average, and opal flux from TT013-PC72 in the equatorial Pacific^33^ (black) over the last two glacial-interglacial cycles. Terminations are indicated by grey bars. All records are plotted on their individual timescales.
