# Supplementary material for: Strong coupling of Asian Monsoon and Antarctic climates on sub-orbital timescales
Source: Sci Rep. 2016 Sep 8;6:32995. doi: 10.1038/srep32995 (PMC5015120; doi:10.1038/srep32995)
Supplement: Supplementary Information [file srep32995-s1.pdf]

Strong coupling of Asian Monsoon and Antarctic climates on sub-orbital timescales

Shitao Chen[1,2], Yongjin Wang[1,2]*, Hai Cheng[3,4], R. Lawrence Edwards[4], Xianfeng Wang[5], Xinggong Kong[1,6] & Dianbing Liu[1,6]

[1]Key Laboratory of Virtual Geographic Environment, Nanjing Normal University, Nanjing 210023, China.
[2]Jiangsu Center for Collaborative Innovation in Geographic Information Resource Development and Application, Nanjing 210023, China.
[3]Institute of Global Environmental Change, Xi'an Jiaotong University, Xi'an 710049, China.
[4]Department of Geology and Geophysics, University of Minnesota, Minneapolis, Minnesota 55455, USA.
[5]Earth Observatory of Singapore, Nanyang Technological University, Nanyang Avenue 639798, Singapore.
[6]State Key Laboratory Cultivation Base of Geographical Environment Evolution, Jiangsu Province, Nanjing 210023, China

*To whom correspondence should be addressed. E-mail:yjwang@njnu.edu.cn

Supplementary information contains 5 figures and 2 tables.

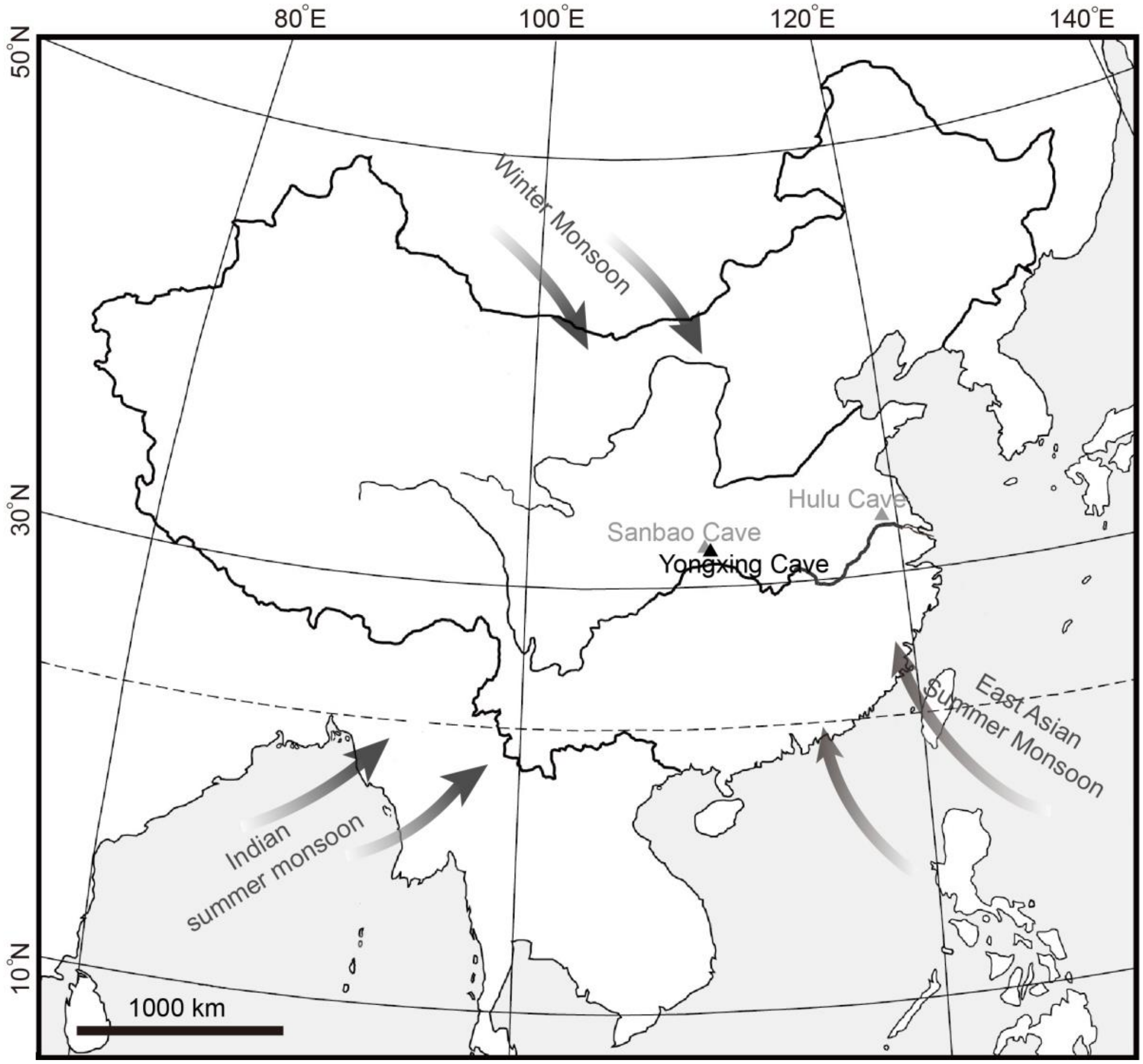

Supplementary Figure S1 | Location of caves. Triangles indicate locations of Yongxing (this study) (31º35′N, 111º14′E), Hulu[14] (32°30′ N, 119°10′E) and Sanbao[4,5] (31º40′N, 110º26′E) caves. The figure was modified from Supplementary Fig. S1 in ref. 46 using the software Photoshop.

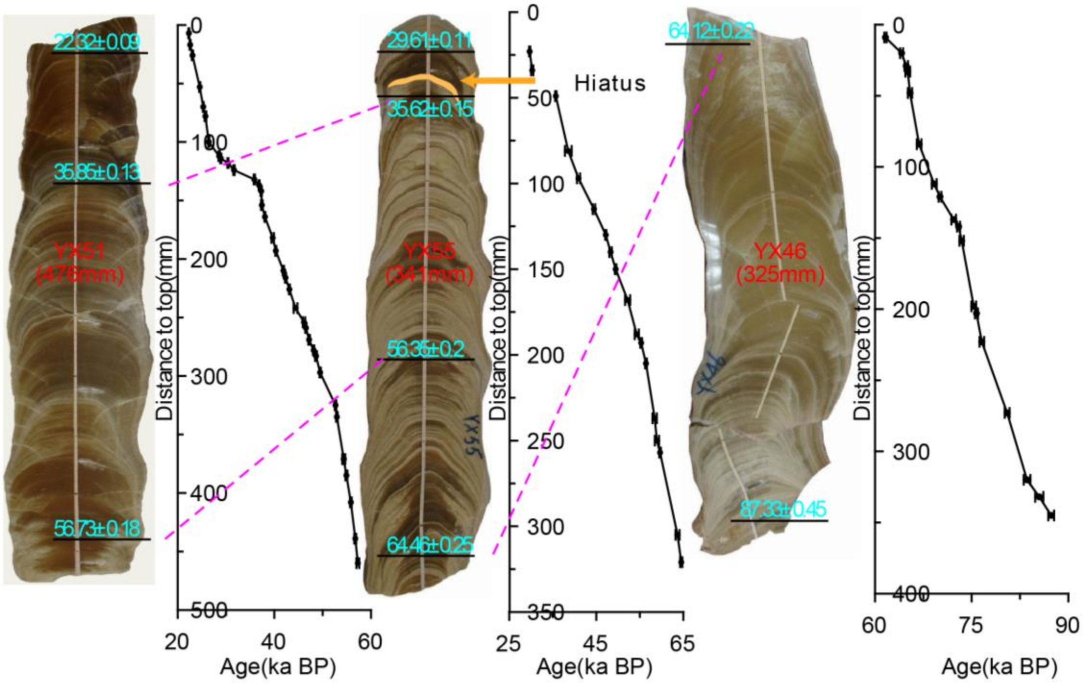

Supplementary Figure S2 | Profiles of stalagmites YX51, YX55, and YX46, and their growth rates. Purple dashed lines indicate contemporaneous growth intervals between the stalagmites, bracketed by the ^{230}Th dates on the polished sections. Sample YX51 exhibits a slow growth rate between 108 and 130 mm, resulting in lower-resolution (~180-260 years) δ^{18}O data between 30 and 35 ka BP. The yellow curved line on YX55 shows a hiatus.

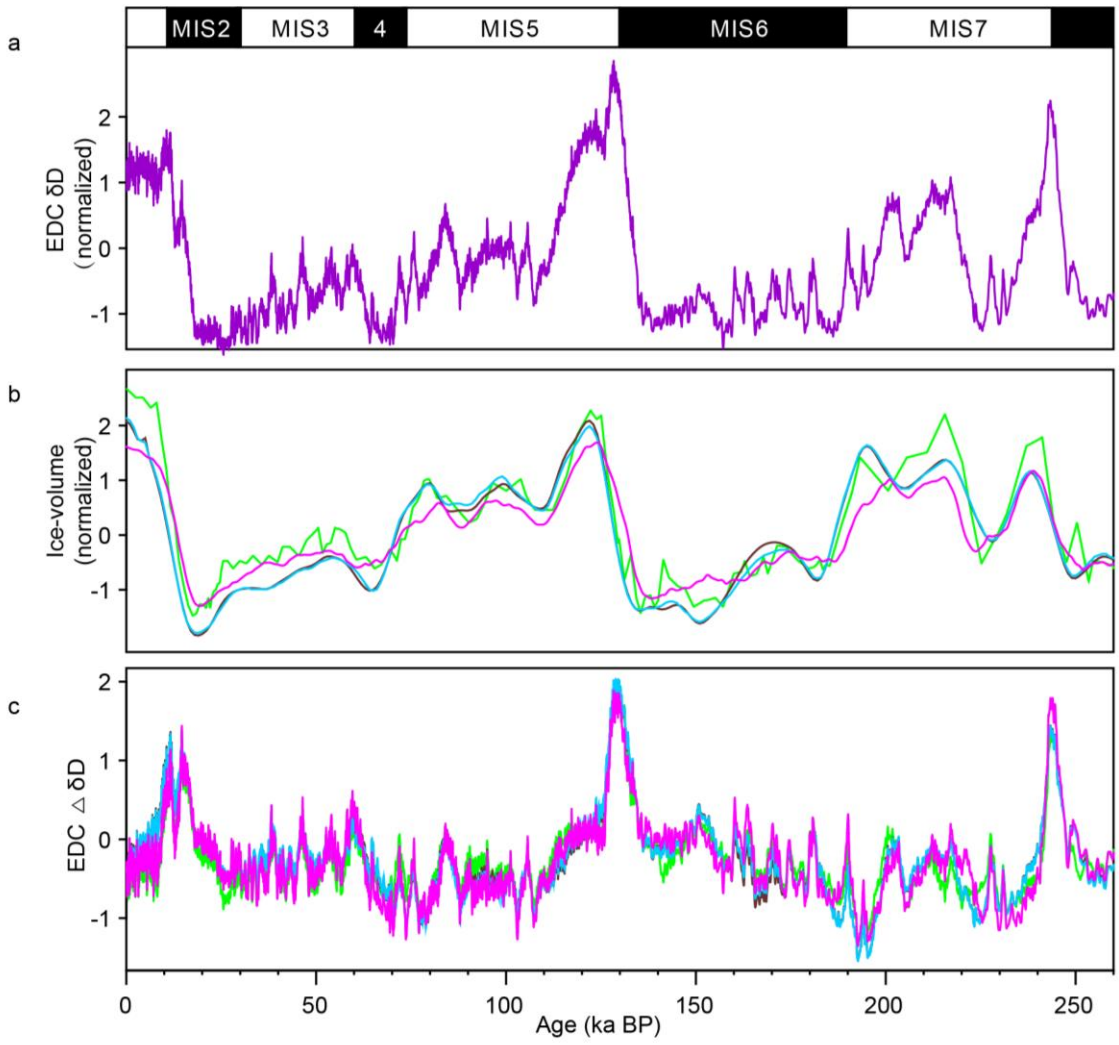

Supplementary Figure S3 | Removal of the ice volume signal from the EDC *δ*D record. a, *δ*D record from the EDC ice core[2] on the AICC2012 chronology[22] (normalized; purple). **b,** Indicators of ice-volume signal. LR04 marine stack[21] (normalized; magenta) and three SPECMAP curves (normalized) initiated by Imbrie *et al*. (1984) (dark brown; ref. 43), Martinson *et al*. (1987) (green; ref. 44), and Imbrie *et al*. (1992) (sky blue; ref. 45), respectively. **c,** Detrended EDC *δ*D record (Δ*δ*D) obtained by subtracting the ice-volume signals in profile **b**, respectively.

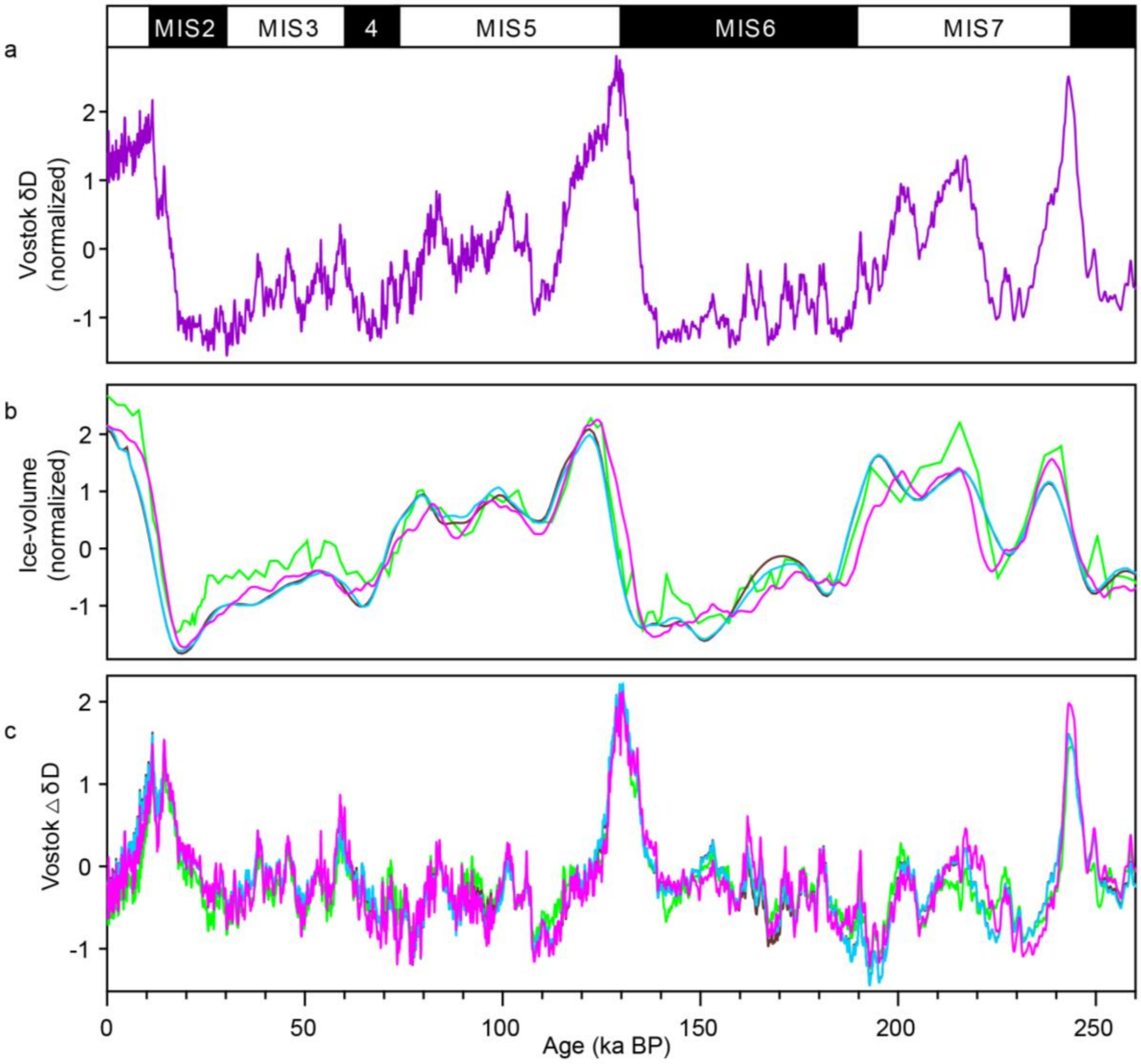

Supplementary Figure S4 | Removal of the ice volume signal from the Vostok δD record[3] following the procedure described in Supplementary Fig. S3.

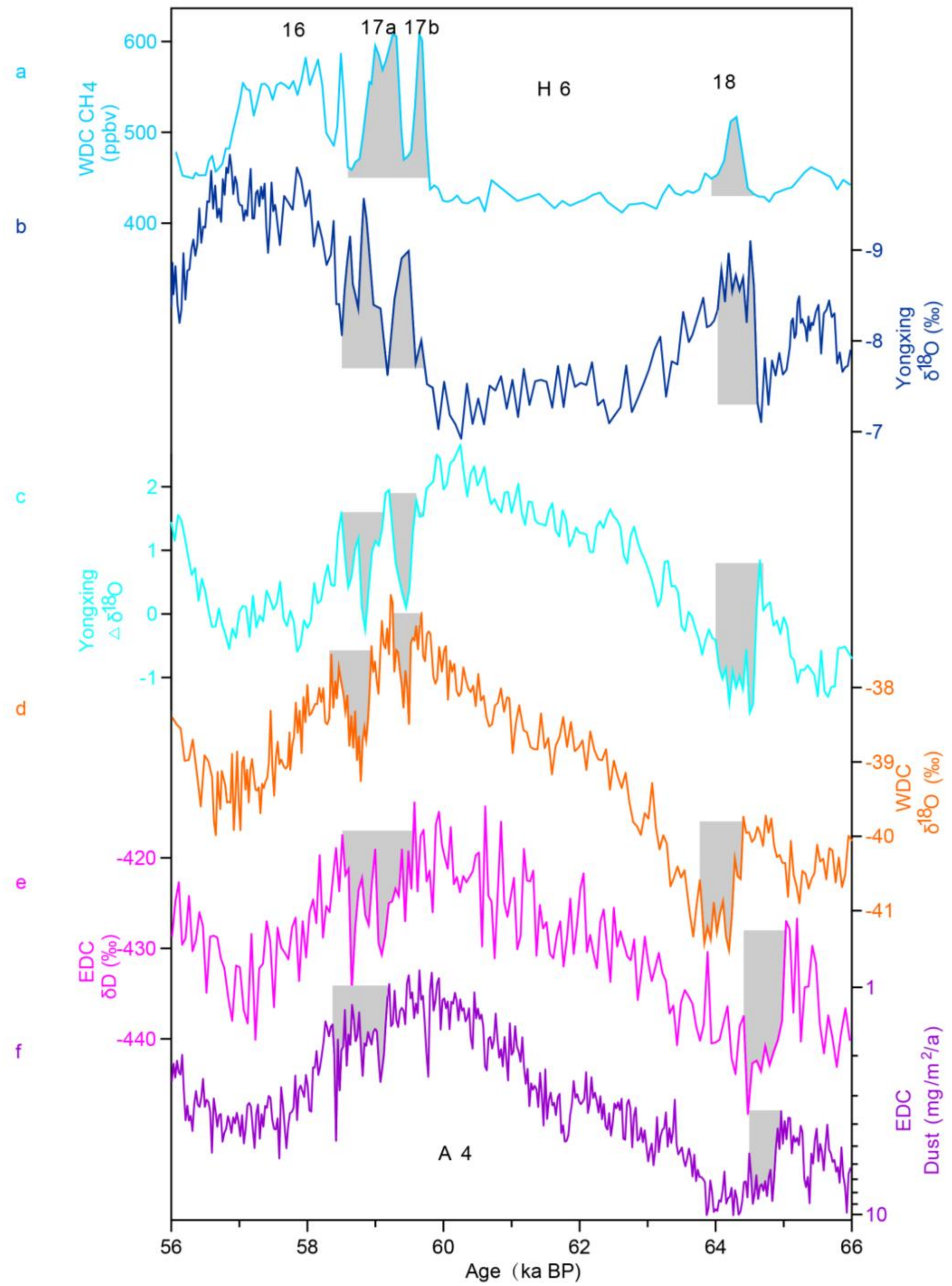

Supplementary Figure S5 | Detailed comparison between AM and Antarctic ice core records from 66 to 56 ka BP. a, WDC methane record[12]. **b,** Yongxing δ^{18}O record. **c,** Detrended Yongxing δ^{18}O record ($\Delta\delta^{18}$O). **d,** WDC δ^{18}O record[12]**. e,** EDC δD record[2]. **f,** EDC dust record[30]. All Antarctic records are plotted on the WD2014 chronology[12]. Short-lived events (DO 18, 17) in the Yongxing record, indicated by grey shading, have counterparts in the Antarctic records.

Supplementary Table S1. ^{230}Th dating results of three stalagmites from Yongxing Cave

Sample	^{238}U (ppb)		^{232}Th (ppt)		^{234}U (measured)		$[^{230}Th/^{238}U]$ (actiivity)		Age (y) (uncorrected)		$^{234}U_{Initial}$ (corrected)		Age (yBP) (corrected)	
**YX51-7**	872.0	± 1.4	2956.1	± 35.6	546.6	±1.8	0.2908	±0.0009	22440	± 80	582.2	± 1.9	**22320**	**±90**
**YX51-17**	915.0	± 2.2	378.2	± 2.2	508.6	±2.2	0.2855	±0.0011	22640	± 110	542.1	± 2.3	**22580**	**±110**
**YX51-26**	953.5	± 2.2	450.5	± 3.8	547.2	±2.6	0.2987	±0.0013	23130	± 120	584.2	± 2.8	**23060**	**±120**
**YX51-53**	943.6	± 2.1	271.6	± 3.5	489.6	±2.5	0.3042	±0.0013	24630	± 120	524.9	± 2.7	**24570**	**±120**
**YX51-70**	803.6	± 1.1	285.3	± 15.5	511.7	±1.5	0.3173	±0.0009	25350	± 80	549.6	± 1.6	**25290**	**±80**
**YX51-78**	683.6	± 2.5	110.2	± 2.3	531.5	±3.8	0.3263	±0.0018	25790	± 170	571.7	± 4.1	**25730**	**±170**
**YX51-100**	834.1	± 1.6	215.7	± 16.7	527.4	±2.3	0.3335	±0.0011	26480	± 110	568.4	± 2.5	**26420**	**±110**
**YX51-112**	652.0	± 1.0	111.8	± 3.5	588.0	±2.1	0.3716	±0.0015	28630	± 140	637.6	± 2.3	**28570**	**±140**
**YX51-114**	749.7	± 1.3	266.8	± 14.1	597.0	±2.2	0.3782	±0.0011	28970	± 110	647.8	± 2.4	**28910**	**±110**
**YX51-118**	354.4	± 0.6	919.1	± 15.4	662.6	±2.3	0.4124	±0.0012	30490	± 110	722.0	± 2.5	**30390**	**±110**
**YX51-124**	340.4	± 0.4	791.4	± 16.7	659.7	±1.6	0.4265	±0.0015	31740	± 140	721.4	± 1.8	**31640**	**±140**
**YX51-132**	383.8	± 0.4	1373.7	± 18.6	669.9	±1.5	0.4785	±0.0013	35970	± 120	741.3	± 1.7	**35850**	**±130**
**YX51-137**	532.0	± 0.7	117.8	± 9.8	565.1	±1.7	0.4580	±0.0008	36910	± 90	627.2	± 1.9	**36850**	**±90**
**YX51-142**	750.6	± 1.6	615.9	± 13.2	561.4	±2.1	0.4615	±0.0012	37350	± 130	623.9	± 2.3	**37270**	**±130**
**YX51-154**	860.8	± 1.7	361.4	± 3.6	545.1	±2.3	0.4570	±0.0016	37470	± 170	606.0	± 2.6	**37410**	**±170**
**YX51-164**	588.9	± 0.8	355.6	± 12.5	579.4	±1.7	0.4752	±0.0008	38110	± 90	645.3	± 1.9	**38050**	**±90**
**YX51-182**	541.2	± 1.3	70.7	± 3.7	602.3	±3.0	0.4988	±0.0025	39710	± 250	673.9	± 3.4	**39650**	**±250**
**YX51-193**	637.5	± 0.8	160.8	± 14.7	597.6	±1.6	0.5044	±0.0011	40360	± 110	669.7	± 1.8	**40290**	**±110**
**YX51-210**	793.1	± 1.1	191.8	± 13.5	510.8	±1.4	0.4916	±0.0011	41920	± 130	575.0	± 1.6	**41860**	**±130**
**YX51-216**	589.3	± 0.6	364.2	± 15.7	652.5	±1.6	0.5443	±0.0011	42380	± 110	735.3	± 1.8	**42310**	**±110**

Continue to next page

Table S1 (Cont.)

Sample	^{238}U (ppb)		^{232}Th (ppt)		^{234}U (measured)		$[^{230}Th/^{238}U]$ (actiivity)		Age (y) (uncorrected)		$^{234}U_{Initial}$ (corrected)		Age (yBP) (corrected)	
**YX51-226**	731.0	± 0.9	105.8	± 12.1	582.9	±1.3	0.5287	±0.0011	43170	± 120	658.4	± 1.5	**43110**	**±120**
**YX51-242**	486.3	± 2.4	145.1	± 1.9	610.2	±6.5	0.5509	±0.0033	44440	± 390	691.9	± 7.4	**44380**	**±390**
**YX51-254**	722.3	± 1.6	204.0	± 2.1	455.7	±2.2	0.5120	±0.0021	46180	± 240	519.1	± 2.5	**46120**	**±240**
**YX51-259**	744.6	± 1.1	260.9	± 10.0	527.4	±1.6	0.5432	±0.0011	46590	± 130	601.6	± 1.9	**46530**	**±130**
**YX51-269**	684.9	± 1.4	245.5	± 13.7	576.0	±2.3	0.5676	±0.0016	47240	± 180	658.2	± 2.6	**47180**	**±180**
**YX51-278**	641.4	± 0.9	174.0	± 9.6	586.0	±1.6	0.5807	±0.0010	48190	± 120	671.5	± 1.9	**48130**	**±120**
**YX51-283**	630.8	± 1.2	145.9	± 12.3	583.7	±2.1	0.5847	±0.0018	48690	± 200	669.8	± 2.4	**48620**	**±200**
**YX51-297**	559.0	± 0.6	241.8	± 13.2	581.4	±1.5	0.5917	±0.0012	49540	± 140	668.6	± 1.8	**49470**	**±140**
**YX51-325**	478.4	± 0.5	241.7	± 14.5	463.7	±1.4	0.5738	±0.0012	52710	± 150	538.0	± 1.6	**52640**	**±150**
**YX51-335**	654.0	± 0.9	263.8	± 13.5	449.2	±1.4	0.5702	±0.0013	52990	± 170	521.6	± 1.7	**52920**	**±170**
**YX51-371**	737.1	±0.9	264	±14	468.6	±1.4	0.5908	±0.0012	54430	±150	546	±2	**54370**	**±150**
**YX51-371**	725.0	± 1.8	294.4	± 2.1	469.6	±2.4	0.5904	±0.0025	54410	± 320	547.5	± 2.8	**54350**	**±320**
**YX51-385**	587.0	± 0.7	168.4	± 14.7	471.3	±1.4	0.5965	±0.0014	54970	± 170	550.3	± 1.7	**54900**	**±170**
**YX51-408**	640.2	± 0.8	309.9	± 13.5	463.6	±1.4	0.6011	±0.0012	55900	± 160	542.8	± 1.7	**55830**	**±160**
**YX51-439**	591.2	± 0.8	177.3	± 12.5	474.5	±1.6	0.6135	±0.0014	56800	± 180	556.9	± 1.9	**56730**	**±180**
**YX51-460**	668.1	± 1.5	506.7	± 2.2	483.1	±2.2	0.6212	±0.0023	57340	± 290	567.9	± 2.7	**57270**	**±290**

Continue to next page

Table S1 (Cont.)

Sample	^{238}U (ppb)		^{232}Th (ppt)		^{234}U (measured)		$[^{230}Th/^{238}U]$ (actiivity)		Age (y) (uncorrected)		$^{234}U_{Initial}$ (corrected)		Age (yBP) (corrected)	
**YX55-23**	469.6	± 0.5	990.0	± 21.5	353.3	±1.3	0.3263	±0.0010	29710	± 110	384.0	± 1.4	**29610**	**±110**
**YX55-34**	472.0	± 1.1	698.9	± 3.5	315.0	±2.6	0.3217	±0.0022	30290	± 250	343.1	± 2.8	**30200**	**±250**
**YX55-49**	357.1	± 0.4	759.1	± 21.0	327.3	±1.4	0.3753	±0.0012	35730	± 150	362.0	± 1.6	**35620**	**±150**
**YX55-81**	419.1	± 1.1	16635.6	± 91.3	416.2	±3.6	0.4351	±0.0059	39330	± 650	464.1	± 4.1	**38480**	**±750**
**YX55-97**	485.3	± 1.5	6209.4	± 19.3	377.7	±4.0	0.4393	±0.0027	41160	± 330	424.0	± 4.5	**40840**	**±360**
**YX55-115**	361.1	± 0.4	6152.7	± 64.6	388.3	±1.5	0.4758	±0.0013	44800	± 160	440.2	± 1.7	**44400**	**±290**
**YX55-130**	744.7	± 1.0	1654.4	± 22.7	328.3	±1.4	0.4746	±0.0008	47220	± 120	375.1	± 1.7	**47110**	**±120**
**YX55-140**	581.1	± 1.2	1964.3	± 6.2	386.8	±2.2	0.5053	±0.0022	48350	± 280	443.2	± 2.6	**48220**	**±290**
**YX55-150**	468.2	± 0.7	3327.5	± 36.7	402.7	±1.7	0.5229	±0.0010	49590	± 140	463.2	± 1.9	**49390**	**±170**
**YX55-168**	340.5	± 0.4	13049.9	± 132.0	387.3	±1.3	0.5445	±0.0013	52960	± 170	448.8	± 1.7	**52130**	**±570**
**YX55-188**	588.1	± 2.3	2169.8	± 9.4	421.0	±4.8	0.5694	±0.0034	54350	± 480	490.8	± 5.6	**54220**	**±480**
**YX55-193**	485.9	± 0.6	1596.0	± 25.6	389.4	±1.5	0.5648	±0.0014	55370	± 190	455.2	± 1.8	**55240**	**±190**
**YX55-205**	445.0	± 0.6	1819.0	± 26.1	403.4	±1.4	0.5799	±0.0014	56490	± 190	473.0	± 1.7	**56350**	**±200**
**YX55-237**	574.5	± 2.1	2671.0	± 9.9	367.5	±4.6	0.5793	±0.0032	58500	± 490	433.4	± 5.4	**58350**	**±490**
**YX55-250**	689.5	± 2.5	1928.5	± 8.1	366.0	±4.2	0.5823	±0.0032	58980	± 480	432.3	± 5.0	**58870**	**±480**
**YX55-257**	737.9	± 0.9	2229.6	± 28.1	386.2	±1.4	0.5976	±0.0012	59710	± 170	456.9	± 1.6	**59590**	**±180**
**YX55-305**	384.5	± 1.5	5623.8	± 33.3	327.3	±2.1	0.6010	±0.0028	63970	± 430	391.7	± 2.5	**63610**	**±450**
**YX55-321**	626.3	± 1.1	3559.0	± 39.1	384.5	±1.7	0.6345	±0.0016	64630	± 240	461.4	± 2.0	**64460**	**±250**

Continue to next page

Table S1 (Cont.)

Sample	^{238}U (ppb)		^{232}Th (ppt)		^{234}U (measured)		$[^{230}Th/^{238}U]$ (actiivity)		Age (y) (uncorrected)		$^{234}U_{Initial}$ (corrected)		Age (yBP) (corrected)	
**YX46-9**	612.9	± 0.7	2664.8	± 30.8	584.5	±1.5	0.7062	±0.0013	61750	± 170	695.7	± 1.9	**61620**	**±180**
**YX46-20**	650.4	± 0.9	110.1	± 16.0	542.2	±1.8	0.7070	±0.0017	64180	± 220	649.8	± 2.2	**64120**	**±220**
**YX46-29**	743.4	± 1.0	196.2	± 14.0	547.0	±3.0	0.7154	±0.0017	64910	± 270	657.0	± 3.7	**64850**	**±270**
**YX46-33**	713.0	± 1.7	468.9	± 3.1	527.7	±2.8	0.7074	±0.0026	65160	± 360	634.4	± 3.4	**65090**	**±360**
**YX46-48**	694.4	± 1.5	386.1	± 2.5	551.4	±2.3	0.7218	±0.0025	65500	± 330	663.2	± 2.8	**65430**	**±330**
**YX46-84**	717.6	± 1.5	57.1	± 3.6	510.2	±2.6	0.7129	±0.0026	66900	± 360	616.4	± 3.3	**66840**	**±360**
**YX46-112**	865.1	± 2.0	107.5	± 3.2	484.5	±2.6	0.7183	±0.0026	69240	± 380	589.2	± 3.2	**69180**	**±380**
**YX46-121**	777.0	± 1.1	36.2	± 11.4	454.3	±2.1	0.7107	±0.0012	70140	± 220	553.9	± 2.5	**70080**	**±220**
**YX46-137**	877.7	± 1.6	142.6	± 3.4	458.7	±2.0	0.7276	±0.0024	72260	± 350	562.7	± 2.5	**72200**	**±350**
**YX46-142**	633.4	± 0.7	90.2	± 12.9	545.6	±2.0	0.7806	±0.0014	73040	± 230	670.4	± 2.5	**72970**	**±230**
**YX46-152**	528.1	± 0.9	134.1	± 2.7	528.6	±2.2	0.7745	±0.0023	73510	± 340	650.7	± 2.8	**73450**	**±340**
**YX46-198**	590.2	± 1.1	1670.9	± 4.6	481.5	±2.3	0.7632	±0.0023	75430	± 360	595.9	± 2.9	**75320**	**±360**
**YX46-203**	569.9	± 0.7	182.8	± 15.0	408.5	±1.4	0.7264	±0.0014	75810	± 230	505.9	± 1.8	**75740**	**±230**
**YX46-223**	523.8	± 0.8	1473.1	± 4.4	544.5	±2.1	0.8074	±0.0025	76680	± 360	676.2	± 2.7	**76580**	**±360**
**YX46-273**	535.8	± 0.9	1742.1	± 4.9	577.8	±2.2	0.8562	±0.0025	80620	± 370	725.6	± 2.9	**80510**	**±370**
**YX46-320**	617.0	± 1.3	5190.2	± 14.3	499.4	±2.2	0.8332	±0.0033	83790	± 510	632.3	± 3.0	**83590**	**±520**
**YX46-332**	618.5	± 0.7	27270.3	± 275.0	499.1	±1.5	0.8511	±0.0016	86320	± 270	635.4	± 2.2	**85480**	**±620**
**YX46-345**	534.3	± 0.9	2529.1	± 6.4	528.6	±2.1	0.8763	±0.0028	87470	± 450	676.7	± 2.8	**87330**	**±450**

The numbers behind the hyphen in the first column show the distance from the top (in mm).

Errors are 2σ analytical errors. Decay constant values are $\lambda_{230}=9.1577\times10^{-6}$ y^{-1}, $\lambda_{234}=2.8263\times10^{-6}$ yr^{-1}, $\lambda_{238}=1.55125\times10^{-10}$ y^{-1}.

Corrected ^{230}Th ages assume an initial $^{230}Th/^{232}Th$ atomic ratio of $(4.4\pm2.2)\times10^{-6}$.

Corrected ^{230}Th ages are indicated in bold. B.P. stands for “Before Present” where the “Present” is defined as the year 1950 A.D.

Supplementary Table S2. Oxygen isotopic data of three stalagmites from Yongxing Cave

Distance (mm)	Age (kyBP)	$\delta^{18}O$ (VPDB)	Distance (mm)	Age (kyBP)	$\delta^{18}O$ (VPDB)	Distance (mm)	Age (kyBP)	$\delta^{18}O$ (VPDB)	Distance (mm)	Age (kyBP)	$\delta^{18}O$ (VPDB)	Distance (mm)	Age (kyBP)	$\delta^{18}O$ (VPDB)
							**YX51**							
4	22.24	-7.06	16	22.55	-7.95	25	23.01	-7.54	34	23.51	-6.88	52	24.51	-7.92
5	22.26	-6.96	16.5	22.56	-8.02	25.5	23.04	-7.85	35	23.56	-6.93	53	24.57	-7.56
6	22.29	-7.26	17	22.58	-7.86	26	23.06	-7.58	36	23.62	-6.47	54	24.61	-7.65
7	22.32	-6.87	17.5	22.60	-8.08	26.5	23.09	-7.92	37	23.68	-6.87	55	24.65	-7.68
8	22.34	-6.98	18	22.63	-7.81	27	23.12	-7.48	38	23.73	-6.40	56	24.69	-7.63
9	22.37	-6.99	18.5	22.66	-8.21	27.5	23.15	-8.03	39	23.79	-5.91	57	24.73	-7.15
10	22.39	-7.38	19	22.68	-7.78	28	23.18	-7.88	40	23.84	-6.23	58	24.78	-7.16
10.5	22.41	-7.68	19.5	22.71	-8.23	28.5	23.20	-8.15	41	23.90	-6.10	59	24.82	-7.15
11	22.42	-7.17	20	22.74	-7.62	29	23.23	-7.96	42	23.95	-6.22	60	24.86	-7.32
11.5	22.43	-7.37	20.5	22.77	-7.70	29.5	23.26	-7.95	43	24.01	-6.26	61	24.90	-7.27
12	22.45	-7.34	21	22.79	-7.37	30	23.29	-7.68	44	24.06	-6.40	62	24.95	-7.45
12.5	22.46	-7.43	21.5	22.82	-7.09	30.5	23.31	-7.11	45	24.12	-6.53	63	24.99	-7.30
13	22.47	-7.27	22	22.85	-6.93	31	23.34	-6.91	46	24.18	-6.65	64	25.03	-7.67
13.5	22.48	-7.32	22.5	22.87	-7.46	31.5	23.37	-7.51	47	24.23	-6.68	65	25.07	-7.29
14	22.50	-7.90	23	22.90	-6.98	32	23.40	-7.07	48	24.29	-7.58	66	25.12	-7.62
14.5	22.51	-7.80	23.5	22.93	-7.29	32.5	23.43	-7.35	49	24.34	-7.41	67	25.16	-7.50
15	22.52	-7.52	24	22.96	-7.32	33	23.45	-6.76	50	24.40	-7.32	68	25.20	-7.60
15.5	22.54	-7.99	24.5	22.98	-7.56	33.5	23.48	-7.10	51	24.45	-7.60	69	25.24	-7.28

Continue to next page

Table S2 (Cont.)

Distance (mm)	Age (kyBP)	$\delta^{18}O$ (VPDB)	Distance (mm)	Age (kyBP)	$\delta^{18}O$ (VPDB)	Distance (mm)	Age (kyBP)	$\delta^{18}O$ (VPDB)	Distance (mm)	Age (kyBP)	$\delta^{18}O$ (VPDB)	Distance (mm)	Age (kyBP)	$\delta^{18}O$ (VPDB)
70	25.29	-7.58	89	26.07	-8.12	104	27.14	-7.76	113.5	28.82	-9.39	123	31.43	-8.52
71	25.34	-7.62	90	26.11	-8.09	104.5	27.23	-7.86	114	28.91	-9.13	123.5	31.54	-8.59
72	25.40	-7.99	91	26.14	-8.26	105	27.32	-7.73	114.5	29.09	-8.77	124	31.64	-8.56
73	25.45	-7.52	92	26.17	-8.45	105.5	27.41	-8.56	115	29.28	-9.15	124.5	31.91	-8.81
74	25.51	-7.48	93	26.20	-8.14	106	27.50	-8.04	115.5	29.46	-8.31	125	32.17	-8.67
75	25.56	-7.21	94	26.23	-8.22	106.5	27.59	-8.46	116	29.65	-7.76	125.5	32.43	-8.72
76	25.62	-6.81	95	26.26	-8.28	107	27.67	-8.10	116.5	29.83	-7.91	126	32.69	-8.04
77	25.67	-7.43	96	26.29	-8.65	107.5	27.76	-8.85	117	30.02	-7.71	126.5	32.96	-8.23
78	25.73	-7.52	97	26.33	-8.49	108	27.85	-8.15	117.5	30.21	-8.02	127	33.22	-7.95
79	25.76	-7.59	98	26.36	-8.40	108.5	27.94	-8.30	118	30.39	-7.32	127.5	33.48	-8.72
80	25.79	-7.75	99	26.39	-8.02	109	28.03	-7.67	118.5	30.50	-7.52	128	33.75	-8.21
81	25.82	-7.57	100	26.42	-8.18	109.5	28.12	-7.80	119	30.60	-7.19	128.5	34.01	-9.00
82	25.85	-7.75	100.5	26.51	-8.16	110	28.21	-7.97	119.5	30.70	-7.56	129	34.27	-8.27
83	25.89	-7.63	101	26.60	-7.92	110.5	28.30	-8.36	120	30.81	-8.06	129.5	34.53	-8.66
84	25.92	-7.79	101.5	26.69	-7.73	111	28.39	-8.62	120.5	30.91	-8.49	130	34.80	-8.33
85	25.95	-7.89	102	26.78	-7.54	111.5	28.48	-9.01	121	31.02	-8.80	130.5	35.06	-9.15
86	25.98	-7.99	102.5	26.87	-7.72	112	28.57	-9.25	121.5	31.12	-8.91	131	35.32	-9.22
87	26.01	-7.67	103	26.96	-7.52	112.5	28.66	-9.59	122	31.23	-8.19	131.5	35.58	-8.51
88	26.04	-8.04	103.5	27.05	-7.92	113	28.74	-9.33	122.5	31.33	-8.70	132	35.85	-8.01

Continue to next page

Table S2 (Cont.)

Distance (mm)	Age (kyBP)	$\delta^{18}O$ (VPDB)	Distance (mm)	Age (kyBP)	$\delta^{18}O$ (VPDB)	Distance (mm)	Age (kyBP)	$\delta^{18}O$ (VPDB)	Distance (mm)	Age (kyBP)	$\delta^{18}O$ (VPDB)	Distance (mm)	Age (kyBP)	$\delta^{18}O$ (VPDB)
132.5	35.95	-8.28	142	37.27	-8.89	160	37.79	-9.19	179	39.38	-6.04	198	40.75	-8.34
133	36.05	-7.82	142.5	37.28	-9.35	161	37.85	-9.06	180	39.47	-6.55	199	40.85	-8.22
133.5	36.15	-8.08	143	37.29	-9.16	162	37.92	-8.86	181	39.56	-7.80	200	40.94	-8.50
134	36.25	-8.34	144	37.30	-9.33	163	37.98	-8.40	182	39.65	-7.25	201	41.03	-8.68
134.5	36.35	-9.10	145	37.31	-9.09	164	38.05	-8.56	183	39.71	-7.82	202	41.12	-8.81
135	36.45	-8.84	146	37.32	-9.13	165	38.14	-8.21	184	39.77	-8.23	203	41.22	-8.61
135.5	36.55	-9.49	147	37.33	-9.31	166	38.23	-8.01	185	39.83	-7.66	204	41.31	-8.50
136	36.65	-8.99	148	37.34	-9.23	167	38.31	-7.88	186	39.89	-7.90	205	41.40	-8.84
136.5	36.75	-9.25	149	37.35	-9.23	168	38.40	-7.50	187	39.94	-7.87	206	41.49	-8.65
137	36.85	-8.85	150	37.36	-9.46	169	38.49	-6.99	188	40.00	-8.22	207	41.58	-8.33
137.5	36.89	-9.33	151	37.37	-9.22	170	38.58	-6.59	189	40.06	-8.35	208	41.68	-7.98
138	36.93	-8.98	152	37.38	-9.13	171	38.67	-7.05	190	40.12	-8.77	209	41.77	-7.38
138.5	36.98	-9.45	153	37.40	-8.70	172	38.76	-7.36	191	40.18	-9.00	210	41.86	-7.75
139	37.02	-9.27	154	37.41	-8.98	173	38.85	-6.96	192	40.24	-8.67	211	41.93	-7.80
139.5	37.06	-9.42	155	37.47	-8.78	174	38.94	-6.66	193	40.29	-8.02	212	42.01	-7.60
140	37.10	-9.45	156	37.53	-9.16	175	39.03	-6.65	194	40.39	-7.92	213	42.08	-7.63
140.5	37.15	-9.56	157	37.60	-9.28	176	39.12	-6.93	195	40.48	-7.79	214	42.16	-7.71
141	37.19	-9.26	158	37.66	-9.22	177	39.21	-6.69	196	40.57	-8.32	215	42.23	-8.11
141.5	37.23	-9.26	159	37.73	-9.13	178	39.30	-6.38	197	40.66	-8.18	216	42.31	-8.39

Continue to next page

Table S2 (Cont.)

Distance (mm)	Age (kyBP)	$\delta^{18}O$ (VPDB)	Distance (mm)	Age (kyBP)	$\delta^{18}O$ (VPDB)	Distance (mm)	Age (kyBP)	$\delta^{18}O$ (VPDB)	Distance (mm)	Age (kyBP)	$\delta^{18}O$ (VPDB)	Distance (mm)	Age (kyBP)	$\delta^{18}O$ (VPDB)
217	42.39	-8.47	236	43.90	-8.19	255	46.20	-8.33	274	47.70	-6.78	288	48.93	-9.05
218	42.47	-8.74	237	43.98	-7.15	256	46.28	-8.58	275	47.81	-6.92	288.5	48.96	-9.34
219	42.55	-8.77	238	44.06	-7.47	257	46.36	-8.26	276	47.91	-6.96	289	48.99	-9.15
220	42.63	-8.60	239	44.14	-7.91	258	46.45	-8.45	277	48.02	-6.88	289.5	49.02	-9.13
221	42.71	-8.75	240	44.22	-8.03	259	46.53	-8.50	278	48.13	-6.71	290	49.05	-8.89
222	42.79	-8.94	241	44.30	-8.58	260	46.59	-8.27	279	48.22	-6.89	290.5	49.08	-8.89
223	42.87	-8.98	242	44.38	-8.73	261	46.66	-8.09	280	48.32	-7.70	291	49.11	-8.73
224	42.95	-9.19	243	44.52	-8.21	262	46.72	-8.26	281	48.42	-7.84	291.5	49.14	-8.90
225	43.03	-8.96	244	44.67	-8.40	263	46.79	-8.46	282	48.52	-7.98	292	49.17	-8.81
226	43.11	-8.48	245	44.81	-8.36	264	46.85	-8.35	282.5	48.57	-8.55	292.5	49.20	-8.77
227	43.19	-8.59	246	44.96	-8.17	265	46.92	-7.91	283	48.62	-8.35	293	49.23	-8.64
228	43.27	-8.59	247	45.10	-8.19	266	46.98	-7.34	283.5	48.65	-8.67	293.5	49.26	-8.98
229	43.35	-8.47	248	45.25	-8.70	267	47.05	-6.91	284	48.68	-8.57	294	49.29	-9.16
230	43.42	-8.64	249	45.39	-8.41	268	47.11	-7.06	284.5	48.71	-8.36	294.5	49.32	-9.12
231	43.50	-8.15	250	45.54	-8.28	269	47.17	-7.02	285	48.74	-8.67	295	49.35	-8.91
232	43.58	-8.52	251	45.68	-8.47	270	47.28	-6.90	285.5	48.77	-8.43	295.5	49.38	-9.24
233	43.66	-7.38	252	45.83	-8.55	271	47.39	-6.63	286	48.80	-8.67	296	49.41	-8.94
234	43.74	-6.89	253	45.97	-8.75	272	47.49	-6.77	286.5	48.84	-8.84	296.5	49.44	-8.85
235	43.82	-7.49	254	46.12	-8.44	273	47.60	-6.62	287	48.87	-8.48	297	49.47	-8.49

Continue to next page

Table S2 (Cont.)

Distance (mm)	Age (kyBP)	$\delta^{18}O$ (VPDB)	Distance (mm)	Age (kyBP)	$\delta^{18}O$ (VPDB)	Distance (mm)	Age (kyBP)	$\delta^{18}O$ (VPDB)	Distance (mm)	Age (kyBP)	$\delta^{18}O$ (VPDB)	Distance (mm)	Age (kyBP)	$\delta^{18}O$ (VPDB)
297.5	49.53	-8.65	310	50.94	-9.14	329	52.75	-9.64	348	53.44	-9.13	367	54.21	-8.76
298	49.58	-8.62	311	51.06	-9.16	330	52.78	-9.57	349	53.48	-9.27	368	54.25	-8.25
298.5	49.64	-8.81	312	51.17	-9.11	331	52.81	-9.49	350	53.52	-9.27	369	54.29	-7.72
299	49.70	-8.63	313	51.28	-9.08	332	52.84	-9.45	351	53.56	-9.40	370	54.33	-7.86
299.5	49.75	-8.67	314	51.40	-9.03	333	52.87	-9.07	352	53.60	-9.13	371	54.37	-7.86
300	49.81	-8.69	315	51.51	-9.21	334	52.89	-9.02	353	53.64	-9.48	372	54.41	-8.15
300.5	49.87	-9.07	316	51.62	-9.38	335	52.92	-8.82	354	53.69	-9.16	373	54.44	-7.74
301	49.92	-8.73	317	51.74	-9.03	336	52.96	-9.01	355	53.73	-9.03	374	54.48	-7.68
301.5	49.98	-9.03	318	51.85	-9.14	337	53.00	-9.00	356	53.77	-9.34	374.5	54.50	-7.99
302	50.04	-8.73	319	51.96	-9.15	338	53.04	-9.06	357	53.81	-9.05	375	54.52	-7.73
302.5	50.09	-9.13	320	52.08	-9.56	339	53.08	-9.28	358	53.85	-9.08	375.5	54.54	-8.55
303	50.15	-8.86	321	52.19	-9.23	340	53.12	-9.37	359	53.89	-9.13	376	54.56	-7.95
303.5	50.21	-9.05	322	52.30	-9.11	341	53.16	-9.29	360	53.93	-9.46	376.5	54.58	-8.18
304	50.26	-8.87	323	52.42	-9.06	342	53.20	-9.04	361	53.97	-9.27	377	54.60	-7.86
305	50.38	-8.55	324	52.53	-9.68	343	53.24	-9.44	362	54.01	-8.86	377.5	54.62	-8.23
306	50.49	-9.01	325	52.64	-9.61	344	53.28	-9.90	363	54.05	-8.78	378	54.64	-8.21
307	50.60	-9.36	326	52.67	-9.72	345	53.32	-9.68	364	54.09	-9.04	378.5	54.65	-8.46
308	50.72	-9.23	327	52.70	-9.61	346	53.36	-9.48	365	54.13	-9.16	379	54.67	-7.95
309	50.83	-8.97	328	52.73	-9.58	347	53.40	-9.32	366	54.17	-9.03	379.5	54.69	-8.35

Continue to next page

Table S2 (Cont.)

Distance (mm)	Age (kyBP)	$\delta^{18}O$ (VPDB)	Distance (mm)	Age (kyBP)	$\delta^{18}O$ (VPDB)	Distance (mm)	Age (kyBP)	$\delta^{18}O$ (VPDB)	Distance (mm)	Age (kyBP)	$\delta^{18}O$ (VPDB)	Distance (mm)	Age (kyBP)	$\delta^{18}O$ (VPDB)
380	54.71	-8.17	390	55.10	-8.66	401	55.55	-9.56	411	55.92	-8.50	426	56.35	-8.94
380.5	54.73	-8.18	390.5	55.12	-8.99	401.5	55.57	-9.87	411.5	55.93	-8.57	427	56.38	-9.07
381	54.75	-8.15	391	55.14	-8.40	402	55.59	-8.95	412	55.95	-8.54	428	56.41	-9.46
381.5	54.77	-8.79	391.5	55.16	-8.70	402.5	55.61	-9.36	412.5	55.96	-8.93	429	56.44	-9.08
382	54.79	-8.62	392	55.18	-8.66	403	55.63	-8.95	413	55.97	-8.48	430	56.47	-9.26
382.5	54.81	-8.73	393	55.22	-8.64	403.5	55.65	-9.51	413.5	55.99	-8.57	431	56.50	-9.25
383	54.83	-8.53	393.5	55.24	-8.91	404	55.67	-9.45	414	56.00	-8.39	432	56.53	-9.54
383.5	54.84	-8.69	394	55.26	-8.62	404.5	55.69	-9.28	414.5	56.02	-8.86	433	56.56	-9.53
384	54.86	-8.51	394.5	55.28	-8.97	405	55.71	-9.22	415	56.03	-8.51	434	56.59	-9.87
385	54.90	-9.30	395	55.30	-8.85	405.5	55.73	-9.58	416	56.06	-8.82	435	56.62	-9.46
385.5	54.92	-9.80	395.5	55.32	-9.11	406	55.75	-8.73	417	56.09	-8.40	436	56.65	-9.71
386	54.94	-9.17	396	55.34	-8.77	406.5	55.77	-9.17	418	56.12	-8.20	437	56.67	-9.36
386.5	54.96	-9.73	396.5	55.37	-9.09	407	55.79	-8.42	419	56.15	-8.35	438	56.70	-9.46
387	54.98	-8.56	397	55.39	-8.68	407.5	55.81	-8.65	420	56.18	-8.97	439	56.73	-9.31
387.5	55.00	-8.88	397.5	55.41	-8.95	408	55.83	-8.79	421	56.21	-8.46	440	56.76	-9.78
388	55.02	-8.68	398	55.43	-8.91	409	55.86	-8.58	422	56.24	-8.77	441	56.78	-9.61
388.5	55.04	-9.06	398.5	55.45	-9.23	409.5	55.87	-8.80	423	56.27	-8.79	442	56.81	-9.91
389	55.06	-8.91	399	55.47	-9.17	410	55.89	-8.37	424	56.30	-9.01	443	56.83	-9.85
389.5	55.08	-9.10	400	55.51	-9.52	410.5	55.90	-8.69	425	56.32	-9.12	444	56.86	-10.05

Continue to next page

Table S2 (Cont.)

Distance (mm)	Age (kyBP)	$\delta^{18}O$ (VPDB)	Distance (mm)	Age (kyBP)	$\delta^{18}O$ (VPDB)	Distance (mm)	Age (kyBP)	$\delta^{18}O$ (VPDB)	Distance (mm)	Age (kyBP)	$\delta^{18}O$ (VPDB)	Distance (mm)	Age (kyBP)	$\delta^{18}O$ (VPDB)
445	56.89	-9.90	451	57.04	-9.49	457	57.19	-9.35	463	57.35	-9.56	469	57.50	-9.43
446	56.91	-9.62	452	57.06	-9.32	458	57.22	-9.35	464	57.37	-9.73	470	57.52	-9.50
447	56.94	-9.81	453	57.09	-9.44	459	57.24	-9.63	465	57.40	-9.25			
448	56.96	-9.74	454	57.12	-9.38	460	57.27	-9.66	466	57.42	-8.97			
449	56.99	-9.53	455	57.14	-9.65	461	57.29	-9.48	467	57.45	-9.24			
450	57.01	-9.49	456	57.17	-9.89	462	57.32	-9.70	468	57.47	-9.74			
							**YX55**							
40	34.82	-8.78	51	35.80	-8.21	62	36.78	-9.35	73	37.77	-9.17	84	38.92	-6.90
41	34.91	-8.68	52	35.89	-8.67	63	36.87	-9.31	74	37.86	-9.22	85	39.07	-6.06
42	35.00	-9.67	53	35.98	-7.76	64	36.96	-9.25	75	37.94	-9.02	86	39.22	-6.75
43	35.09	-8.99	54	36.07	-8.61	65	37.05	-9.27	76	38.03	-9.19	87	39.36	-6.19
44	35.18	-9.62	55	36.16	-8.65	66	37.14	-9.94	77	38.12	-8.76	88	39.51	-6.56
45	35.26	-9.17	56	36.25	-8.93	67	37.23	-9.70	78	38.21	-9.21	89	39.66	-6.41
46	35.35	-8.51	57	36.34	-8.61	68	37.32	-9.27	79	38.30	-8.78	90	39.81	-6.65
47	35.44	-7.73	58	36.43	-9.07	69	37.41	-9.62	80	38.39	-8.31	91	39.95	-7.85
48	35.53	-8.20	59	36.52	-9.09	70	37.50	-9.73	81	38.48	-7.49	92	40.10	-7.98
49	35.62	-8.64	60	36.60	-8.95	71	37.59	-9.74	82	38.63	-7.06	93	40.25	-8.41
50	35.71	-8.66	61	36.69	-9.05	72	37.68	-8.99	83	38.78	-7.07	94	40.39	-7.87

Continue to next page

Table S2 (Cont.)

Distance (mm)	Age (kyBP)	$\delta^{18}O$ (VPDB)	Distance (mm)	Age (kyBP)	$\delta^{18}O$ (VPDB)	Distance (mm)	Age (kyBP)	$\delta^{18}O$ (VPDB)	Distance (mm)	Age (kyBP)	$\delta^{18}O$ (VPDB)	Distance (mm)	Age (kyBP)	$\delta^{18}O$ (VPDB)
95	40.54	-8.23	114	44.20	-8.25	133	47.45	-6.44	144	48.69	-8.64	153.5	49.92	-9.03
96	40.69	-8.29	115	44.40	-8.71	134	47.56	-6.87	144.5	48.75	-8.81	154	50.00	-8.89
97	40.84	-8.75	116	44.58	-9.15	135	47.67	-6.74	145	48.81	-8.63	154.5	50.08	-9.15
98	41.03	-8.36	117	44.76	-8.32	136	47.78	-7.26	145.5	48.86	-9.03	155	50.15	-8.86
99	41.23	-8.87	118	44.94	-8.64	136.5	47.84	-7.29	146	48.92	-8.82	155.5	50.23	-9.07
100	41.43	-8.32	119	45.12	-8.40	137	47.89	-6.72	146.5	48.98	-8.75	156	50.30	-8.85
101	41.63	-7.68	120	45.31	-8.47	137.5	47.95	-7.13	147	49.04	-8.73	157	50.46	-8.95
102	41.83	-7.93	121	45.49	-8.73	138	48.00	-6.88	147.5	49.10	-8.54	158	50.61	-9.21
103	42.02	-7.94	122	45.67	-8.70	138.5	48.06	-6.70	148	49.16	-8.69	159	50.76	-8.83
104	42.22	-9.11	123	45.85	-8.11	139	48.11	-6.93	148.5	49.21	-9.05	160	50.91	-9.20
105	42.42	-8.44	124	46.03	-8.75	139.5	48.17	-7.03	149	49.27	-8.65	161	51.07	-8.85
106	42.62	-9.10	125	46.21	-8.63	140	48.22	-6.93	149.5	49.33	-8.54	162	51.22	-9.05
107	42.82	-8.75	126	46.39	-8.63	140.5	48.28	-7.32	150	49.39	-8.54	163	51.37	-9.19
108	43.01	-8.59	127	46.57	-8.34	141	48.34	-7.03	150.5	49.47	-8.50	164	51.52	-9.06
109	43.21	-8.52	128	46.75	-8.80	141.5	48.40	-7.97	151	49.54	-8.34	165	51.68	-8.82
110	43.41	-8.22	129	46.93	-7.99	142	48.46	-8.23	151.5	49.62	-8.66	166	51.83	-9.23
111	43.61	-7.57	130	47.11	-7.06	142.5	48.51	-8.31	152	49.69	-8.75	167	51.98	-9.60
112	43.81	-7.78	131	47.22	-7.01	143	48.57	-8.25	152.5	49.77	-9.03	168	52.13	-9.49
113	44.01	-7.42	132	47.34	-6.87	143.5	48.63	-8.50	153	49.85	-8.83	169	52.24	-9.49

Continue to next page

Table S2 (Cont.)

Distance (mm)	Age (kyBP)	$\delta^{18}O$ (VPDB)	Distance (mm)	Age (kyBP)	$\delta^{18}O$ (VPDB)	Distance (mm)	Age (kyBP)	$\delta^{18}O$ (VPDB)	Distance (mm)	Age (kyBP)	$\delta^{18}O$ (VPDB)	Distance (mm)	Age (kyBP)	$\delta^{18}O$ (VPDB)
170	52.34	-9.73	187.5	54.17	-8.17	198	55.70	-8.97	209	56.60	-9.69	228	57.78	-9.44
171	52.45	-9.32	188	54.22	-8.21	198.5	55.75	-9.15	210	56.66	-9.28	229	57.85	-9.91
172	52.55	-9.53	188.5	54.33	-8.18	199	55.80	-8.54	211	56.72	-9.28	230	57.91	-9.78
173	52.66	-9.25	189	54.43	-7.83	199.5	55.84	-8.70	212	56.79	-9.42	231	57.97	-9.48
174	52.76	-9.77	189.5	54.53	-7.61	200	55.89	-8.55	213	56.85	-9.47	232	58.03	-9.66
175	52.86	-9.04	190	54.63	-7.64	200.5	55.93	-8.85	214	56.91	-9.53	233	58.10	-9.04
176	52.97	-9.09	191	54.84	-8.40	201	55.98	-8.22	215	56.97	-9.31	234	58.16	-9.32
177	53.07	-9.00	191.5	54.94	-8.79	201.5	56.03	-8.47	216	57.04	-9.96	235	58.22	-9.12
178	53.18	-9.44	192	55.04	-9.16	202	56.07	-8.20	217	57.10	-9.74	236	58.28	-8.84
179	53.28	-9.08	192.5	55.14	-9.34	202.5	56.12	-8.45	218	57.16	-9.67	237	58.34	-8.95
180	53.39	-9.45	193	55.24	-8.41	203	56.17	-8.11	219	57.22	-9.71	238	58.39	-9.32
181	53.49	-9.09	193.5	55.29	-8.61	203.5	56.21	-8.32	220	57.28	-9.55	239	58.43	-8.40
182	53.60	-9.14	194	55.34	-8.44	204	56.26	-8.56	221	57.35	-9.40	240	58.47	-8.41
183	53.70	-9.13	194.5	55.38	-8.40	204.5	56.30	-8.92	222	57.41	-9.36	241	58.51	-8.07
184	53.81	-9.08	195	55.43	-8.61	205	56.35	-8.73	223	57.47	-9.62	242	58.55	-8.55
185	53.91	-8.51	195.5	55.47	-8.86	205.5	56.38	-9.01	224	57.53	-9.46	243	58.59	-8.88
186	54.01	-9.50	196	55.52	-9.34	206	56.41	-8.62	225	57.60	-9.06	244	58.63	-9.15
186.5	54.07	-9.30	196.5	55.57	-9.53	207	56.47	-9.04	226	57.66	-9.60	245	58.67	-8.63
187	54.12	-8.33	197	55.61	-9.22	208	56.54	-9.21	227	57.72	-9.52	246	58.71	-8.50

Continue to next page

Table S2 (Cont.)

Distance (mm)	Age (kyBP)	$\delta^{18}O$ (VPDB)	Distance (mm)	Age (kyBP)	$\delta^{18}O$ (VPDB)	Distance (mm)	Age (kyBP)	$\delta^{18}O$ (VPDB)	Distance (mm)	Age (kyBP)	$\delta^{18}O$ (VPDB)	Distance (mm)	Age (kyBP)	$\delta^{18}O$ (VPDB)
247	58.75	-8.36	266	60.34	-7.53	285	61.93	-7.49	304	63.52	-8.32	323	64.56	-8.60
248	58.79	-9.01	267	60.43	-7.09	286	62.02	-7.54	305	63.60	-7.99	324	64.62	-7.31
249	58.83	-9.57	268	60.51	-7.37	287	62.10	-7.52	306	63.66	-8.07	325	64.67	-7.11
250	58.87	-9.34	269	60.59	-7.08	288	62.18	-7.77	307	63.71	-8.20	326	64.72	-7.81
251	58.97	-8.40	270	60.68	-7.51	289	62.27	-7.29	308	63.76	-8.34	327	64.77	-7.30
252	59.07	-8.36	271	60.76	-7.45	290	62.35	-7.35	309	63.82	-8.48	328	64.83	-7.79
253	59.18	-7.62	272	60.85	-7.61	291	62.43	-7.09	310	63.87	-8.15	329	64.88	-7.62
254	59.28	-8.46	273	60.93	-7.25	292	62.52	-7.19	311	63.92	-8.17	330	64.93	-7.87
255	59.38	-8.91	274	61.01	-7.57	293	62.60	-7.27	312	63.98	-8.22	331	64.99	-7.63
256	59.49	-8.99	275	61.10	-7.16	294	62.69	-7.73	313	64.03	-8.35	332	65.04	-7.68
257	59.59	-7.75	276	61.18	-7.69	295	62.77	-7.22	314	64.08	-8.78	333	65.09	-7.76
258	59.67	-8.01	277	61.26	-7.30	296	62.85	-7.36	315	64.14	-8.43			
259	59.76	-7.52	278	61.35	-7.56	297	62.94	-7.52	316	64.19	-8.96			
260	59.84	-7.49	279	61.43	-7.57	298	63.02	-7.69	317	64.24	-8.55			
261	59.93	-7.03	280	61.51	-7.55	299	63.10	-7.91	318	64.30	-8.73			
262	60.01	-7.55	281	61.60	-7.26	300	63.19	-8.05	319	64.35	-8.56			
263	60.09	-7.19	282	61.68	-7.72	301	63.27	-7.39	320	64.40	-8.70			
264	60.18	-7.08	283	61.77	-7.27	302	63.35	-7.78	321	64.46	-8.20			
265	60.26	-6.92	284	61.85	-7.74	303	63.44	-7.74	322	64.51	-9.10			

Continue to next page

Table S2 (Cont.)

Distance (mm)	Age (kyBP)	$\delta^{18}O$ (VPDB)	Distance (mm)	Age (kyBP)	$\delta^{18}O$ (VPDB)	Distance (mm)	Age (kyBP)	$\delta^{18}O$ (VPDB)	Distance (mm)	Age (kyBP)	$\delta^{18}O$ (VPDB)	Distance (mm)	Age (kyBP)	$\delta^{18}O$ (VPDB)
							**YX46**							
8	61.39	-7.68	26	64.60	-9.31	44	65.34	-8.00	62	65.98	-7.90	80	66.68	-8.25
9	61.62	-7.74	27	64.68	-8.75	45	65.36	-8.27	63	66.02	-7.80	81	66.72	-7.98
10	61.85	-7.58	28	64.76	-8.19	46	65.39	-8.11	64	66.06	-7.99	82	66.76	-8.32
11	62.07	-8.02	29	64.85	-7.77	47	65.41	-8.20	65	66.10	-7.40	83	66.80	-8.29
12	62.30	-7.89	30	64.91	-8.06	48	65.43	-8.17	66	66.14	-8.08	84	66.84	-8.18
13	62.53	-7.71	31	64.97	-7.51	49	65.47	-8.32	67	66.18	-7.86	85	66.92	-7.92
14	62.76	-7.64	32	65.03	-7.54	50	65.51	-8.40	68	66.21	-8.02	86	67.01	-7.81
15	62.98	-7.34	33	65.09	-7.94	51	65.55	-7.86	69	66.25	-8.09	87	67.09	-7.73
16	63.21	-7.74	34	65.11	-8.21	52	65.59	-8.30	70	66.29	-8.37	88	67.18	-7.63
17	63.44	-8.01	35	65.13	-8.25	53	65.63	-8.34	71	66.33	-8.31	89	67.26	-7.47
18	63.67	-8.33	36	65.16	-8.03	54	65.67	-8.45	72	66.37	-7.80	90	67.34	-7.61
19	63.89	-8.23	37	65.18	-8.08	55	65.71	-8.26	73	66.41	-8.43	91	67.43	-7.70
20	64.12	-8.75	38	65.20	-8.43	56	65.75	-8.30	74	66.45	-8.34	92	67.51	-7.04
21	64.20	-8.44	39	65.23	-8.49	57	65.78	-7.75	75	66.49	-8.16	93	67.59	-7.19
22	64.28	-8.50	40	65.25	-8.20	58	65.82	-7.87	76	66.53	-8.37	94	67.68	-7.28
23	64.36	-8.54	41	65.27	-8.24	59	65.86	-7.66	77	66.57	-8.32	95	67.76	-7.28
24	64.44	-8.87	42	65.29	-8.19	60	65.90	-7.71	78	66.61	-8.55	96	67.84	-7.54
25	64.52	-8.98	43	65.32	-8.15	61	65.94	-7.73	79	66.65	-7.93	97	67.93	-7.48

Continue to next page

Table S2 (Cont.)

Distance (mm)	Age (kyBP)	$\delta^{18}O$ (VPDB)	Distance (mm)	Age (kyBP)	$\delta^{18}O$ (VPDB)	Distance (mm)	Age (kyBP)	$\delta^{18}O$ (VPDB)	Distance (mm)	Age (kyBP)	$\delta^{18}O$ (VPDB)	Distance (mm)	Age (kyBP)	$\delta^{18}O$ (VPDB)
98	68.01	-7.27	117	69.68	-5.96	136	72.07	-6.55	155	73.57	-8.94	174	74.34	-8.73
99	68.10	-7.75	118	69.78	-6.80	137	72.20	-6.60	156	73.61	-8.88	175	74.38	-8.31
100	68.18	-7.00	119	69.88	-6.06	138	72.35	-6.22	157	73.65	-8.94	176	74.42	-8.80
101	68.26	-7.15	120	69.98	-7.25	139	72.51	-5.84	158	73.69	-8.82	177	74.46	-8.54
102	68.35	-7.13	121	70.08	-7.49	140	72.66	-5.30	159	73.73	-8.49	178	74.50	-8.24
103	68.43	-7.47	122	70.22	-7.59	141	72.82	-5.74	160	73.77	-8.99	179	74.55	-9.11
104	68.51	-7.49	123	70.35	-7.32	142	72.97	-5.87	161	73.81	-8.86	180	74.59	-8.95
105	68.60	-7.25	124	70.48	-7.26	143	73.02	-6.21	162	73.85	-9.05	181	74.63	-8.66
106	68.68	-7.14	125	70.61	-7.38	144	73.07	-6.01	163	73.90	-8.95	182	74.67	-8.91
107	68.76	-7.00	126	70.74	-7.19	145	73.12	-6.38	164	73.94	-9.02	183	74.71	-8.79
108	68.85	-7.12	127	70.88	-7.75	146	73.16	-6.69	165	73.98	-8.76	184	74.75	-8.48
109	68.93	-6.84	128	71.01	-7.66	147	73.21	-6.64	166	74.02	-8.94	185	74.79	-9.19
110	69.01	-6.76	129	71.14	-7.46	148	73.26	-7.35	167	74.06	-8.63	186	74.83	-9.08
111	69.10	-7.13	130	71.27	-7.69	149	73.31	-7.48	168	74.10	-8.50	187	74.87	-8.80
112	69.18	-7.82	131	71.41	-7.97	150	73.35	-7.37	169	74.14	-8.54	188	74.91	-9.20
113	69.28	-7.70	132	71.54	-8.31	151	73.40	-8.09	170	74.18	-8.56	189	74.95	-8.76
114	69.38	-7.74	133	71.67	-7.65	152	73.45	-8.27	171	74.22	-8.76	190	74.99	-9.04
115	69.48	-7.54	134	71.80	-8.29	153	73.49	-8.60	172	74.26	-8.68	191	75.03	-8.89
116	69.58	-6.57	135	71.93	-8.14	154	73.53	-9.05	173	74.30	-8.84	192	75.07	-8.87

Continue to next page

Table S2 (Cont.)

Distance (mm)	Age (kyBP)	$\delta^{18}O$ (VPDB)	Distance (mm)	Age (kyBP)	$\delta^{18}O$ (VPDB)	Distance (mm)	Age (kyBP)	$\delta^{18}O$ (VPDB)	Distance (mm)	Age (kyBP)	$\delta^{18}O$ (VPDB)	Distance (mm)	Age (kyBP)	$\delta^{18}O$ (VPDB)
193	75.11	-8.65	212	76.12	-7.57	231	77.21	-9.59	250	78.70	-10.02	269	80.20	-9.90
194	75.16	-8.44	213	76.16	-7.65	232	77.28	-9.81	251	78.78	-10.03	270	80.27	-9.85
195	75.20	-8.75	214	76.20	-7.71	233	77.36	-9.09	252	78.86	-10.42	271	80.35	-10.30
196	75.24	-8.69	215	76.24	-7.65	234	77.44	-9.32	253	78.94	-10.21	272	80.43	-10.25
197	75.28	-8.78	216	76.28	-7.75	235	77.52	-9.65	254	79.02	-9.89	273	80.51	-9.87
198	75.32	-8.62	217	76.33	-7.57	236	77.60	-9.52	255	79.09	-9.91	274	80.58	-10.19
199	75.40	-8.77	218	76.37	-7.21	237	77.68	-9.96	256	79.17	-9.90	275	80.64	-10.02
200	75.49	-8.16	219	76.41	-7.83	238	77.76	-9.91	257	79.25	-9.66	276	80.71	-10.15
201	75.57	-8.11	220	76.45	-7.74	239	77.84	-9.64	258	79.33	-9.64	277	80.77	-9.84
202	75.66	-8.00	221	76.49	-8.27	240	77.91	-9.87	259	79.41	-10.00	278	80.84	-10.16
203	75.74	-7.39	222	76.53	-8.61	241	77.99	-10.16	260	79.49	-10.15	279	80.90	-9.93
204	75.78	-7.30	223	76.58	-9.10	242	78.07	-10.31	261	79.57	-10.18	280	80.97	-9.81
205	75.83	-7.38	224	76.65	-9.44	243	78.15	-9.97	262	79.64	-10.20	281	81.03	-9.82
206	75.87	-7.68	225	76.73	-9.47	244	78.23	-9.97	263	79.72	-9.79	282	81.10	-10.46
207	75.91	-7.30	226	76.81	-9.25	245	78.31	-9.89	264	79.80	-10.01	283	81.16	-10.35
208	75.95	-7.19	227	76.89	-9.30	246	78.39	-9.94	265	79.88	-10.11	284	81.23	-10.49
209	75.99	-7.59	228	76.97	-9.40	247	78.46	-9.99	266	79.96	-9.89	285	81.30	-9.90
210	76.03	-7.38	229	77.05	-9.54	248	78.54	-9.80	267	80.04	-9.67	286	81.36	-10.20
211	76.08	-7.84	230	77.13	-9.54	249	78.62	-9.59	268	80.12	-9.88	287	81.43	-10.05

Continue to next page

Table S2 (Cont.)

**Distance (mm)**	**Age (kyBP)**	**$\delta^{18}O$ (VPDB)**	**Distance (mm)**	**Age (kyBP)**	**$\delta^{18}O$ (VPDB)**	**Distance (mm)**	**Age (kyBP)**	**$\delta^{18}O$ (VPDB)**	**Distance (mm)**	**Age (kyBP)**	**$\delta^{18}O$ (VPDB)**	**Distance (mm)**	**Age (kyBP)**	**$\delta^{18}O$ (VPDB)**
288	81.49	-10.23	302	82.41	-10.51	316	83.32	-9.79	334	85.76	-7.40	349	87.90	-7.71
289	81.56	-10.27	303	82.47	-10.02	317	83.39	-10.06	335	85.91	-7.95	350	88.04	-8.56
290	81.62	-9.98	304	82.54	-10.38	318	83.45	-9.58	336	86.05	-8.36	351	88.18	-9.09
291	81.69	-10.21	305	82.60	-10.21	319	83.52	-9.66	337	86.19	-8.17	352	88.33	-8.63
292	81.75	-10.42	306	82.67	-10.16	320	83.59	-9.64	338	86.33	-7.82	353	88.47	-8.99
293	81.82	-9.87	307	82.73	-9.18	321	83.74	-9.98	339	86.48	-8.17	354	88.61	-9.67
294	81.88	-9.72	308	82.80	-9.91	322	83.90	-9.65	340	86.62	-7.76	355	88.75	-8.42
295	81.95	-9.87	309	82.87	-9.60	323	84.06	-9.73	341	86.76	-8.14			
296	82.02	-9.81	310	82.93	-9.74	324	84.22	-9.73	342	86.90	-8.11			
297	82.08	-9.98	311	83.00	-9.64	325	84.37	-9.83	343	87.04	-8.12			
298	82.15	-10.10	312	83.06	-9.80	326	84.53	-9.63	344	87.19	-8.10			
299	82.21	-9.90	313	83.13	-9.79	327	84.69	-9.87	345	87.33	-7.96			
300	82.28	-9.65	314	83.19	-9.56	328	84.85	-9.71	346	87.47	-7.68			
301	82.34	-10.45	315	83.26	-9.65	329	85.01	-9.52	347	87.61	-7.53			
